# Coculture of cancer cells with platelets increases their survival and metastasis by activating the TGFβ/Smad/PAI-1 and PI3K/AKT pathways

**DOI:** 10.7150/ijbs.85986

**Published:** 2023-08-15

**Authors:** Haibo Tong, Koukou Li, Muya Zhou, Renfei Wu, Hongmei Yang, Zheng Peng, Qi Zhao, Kathy Qian Luo

**Affiliations:** 1 Faculty of Health Sciences, University of Macau, Taipa, Macao SAR, China.; 2 Ministry of Education Frontiers Science Center for Precision Oncology, University of Macau.

**Keywords:** Cancer cells, platelets, survival, metastasis, TGFβ, PAI-1

## Abstract

When cancer cells enter the bloodstream, they can interact with platelets to acquire stronger survival and metastatic abilities. To elucidate the underlying mechanisms, we cocultured metastatic melanoma and triple-negative breast cancer cells with species-homologous platelets. We found that cocultured cancer cells displayed higher viabilities in circulation, stronger capacities for cell migration, invasion, and colony formation *in vitro*, and more tumorigenesis and metastasis in mice. RNA sequencing analysis revealed that the level of serpin family E member 1 (SERPINE1) was significantly upregulated in cocultured cancer cells. Knockdown of SERPINE1 reversed the coculture-elevated survival and metastatic phenotypes of cancer cells. Mechanistic studies indicated that coculture with platelets activated the TGFβ/Smad pathway to induce SERPINE1 expression in cancer cells, which encodes plasminogen activator inhibitor 1 (PAI-1). PAI-1 then activated PI3K to increase the phosphorylation of AKT^Thr308^ and Bad to elevate Bcl-2, which enhanced cell survival in circulation. Moreover, higher levels of PAI-1 were detected in metastatic tumors from melanoma and triple-negative breast cancer patients than in normal tissues, and high levels of PAI-1 were associated with a shorter overall survival time and worse disease progression in breast cancer. PAI-1 may act as a potential biomarker for detecting and treating metastatic tumor cells.

## Introduction

When cancer cells enter the bloodstream, they become circulating tumor cells (CTCs), which can be easily destroyed by fluidic shear stress (SS), immune attack and anoikis 1. CTCs can use different approaches to overcome these challenges. They can express high levels of important genes, including manganese superoxide dismutase (MnSOD), desmocollin-2 (DSC2), and plakophilin-1 (PKP1), to enhance their survival in circulation 2, 3. They can also form clusters either by themselves 3 or with blood cells such as erythrocytes, leukocytes or platelets to help them metastasize 4, 5. In this study, we investigated how platelets support the survival of CTCs for the following two reasons. First, when cancer cells transmigrate across the endothelium, the invasion site on the blood well creates a small wound that can subsequently recruit and activate platelets to help tumor cells complete the metastasis cascade. Second, erythrocytes are the most numerous cells in the blood, while platelets are the second most numerous cells in the blood; for perspective, there are more than 30 times more platelets in the blood than white blood cells 6, making platelets more likely to encounter and interact with CTCs.

Platelets are differentiated from megakaryocytes and can circulate in the bloodstream. They mainly function in blood coagulation and wound healing. Hyperactivation of platelets has been observed in some cancers, such as colon cancer, and is involved in cancer progression 7. In addition, thrombocytosis is correlated with worse prognosis, as confirmed in patients with ovarian 8, colorectal 9, gastric 10, lung 11, and breast cancer 12. Although platelets do not have nuclei, they have some organelles, such as lysosomes and vesicles containing α-granules and dense granules 13.

The premise of cancer metastasis is the survival of cancer cells in circulation. Understanding the interactions between platelets and tumor cells and the role of platelets in cancer dissemination are important for studying the mechanisms of metastasis. It has been reported that CTCs can interact with platelets to shield them from tumor necrosis factor (TNF-α) and natural killer (NK) cell-mediated cell death 14, 15. Tumor cells can utilize the major histocompatibility complex class I molecules transferred by platelets to evade immune surveillance 16, or platelet-derived transforming growth factor-β (TGFβ) can downregulate killer cell lectin like receptor K1 (encodes NKG2D) to inhibit the antitumor reactivity of NK cells 17. Platelets can also activate some important genes, such as yes associated protein 1 (YAP), in tumor cells to support their survival in circulation 18. In addition, knockout of receptors on platelets, such as glycoprotein Ib platelet subunit alpha (GPIbα), glycoprotein VI platelet (GPVI), and P-selectin, can reduce cancer cell survival and experimental metastasis 19-22. Platelets can release multiple substances upon activation with biological functions such as protection from shear stress-induced damage, evasion of immune surveillance, and promotion of invasion and metastasis processes 23-28. For example, growth factors or angiogenic factors from α-granules, such as TGFβ, platelet-derived growth factor (PDGF), basic fibroblast growth factor (bFGF), and vascular endothelial growth factor (VEGF), are released when platelets are activated and are involved in every stage of carcinogenesis 25, 27-30. Platelets can also support tumor cell survival and metastasis by inducing an epithelial-mesenchymal transition (EMT) in colon and breast cells 31, which plays an important role in drug resistance and metastasis of tumor cells 32.

In B16F10 melanoma or lung cancer cells, CTC clusters are more likely to complete hematogenous metastasis than single CTCs 33, 34. It is still unclear how CTCs become more metastatic after interacting with platelets. Therefore, in this study, we chose the malignant murine melanoma B16F10-C3 and metastatic human triple-negative breast cancer (TNBC) 231-GFP as the two model cell lines and cocultured them with species-homologous platelets to study their interactions.

Melanoma was among the top five cancers in terms of the estimated number of new cases, and breast cancer was the top-ranked cancer in females in the United States in 2022 35. Melanoma, derived from melanocytes, is regarded as the most severe type of skin cancer. In early stages, it can be cured by surgical treatment. However, advanced melanoma becomes metastatic and has a poor prognosis 36, 37.

Breast cancer is the primary cause of cancer-related deaths in women. Its subtypes include luminal A, luminal B, HER2^+^ and TNBC 38. TNBC constitutes approximately 15% of all breast cancers and has a low 5-year relative survival rate and high metastatic potential 39, 40. For TNBC patients, there is currently no targeted therapy available. In general, malignant cancer cells are more aggressive, have a higher capacity to invade and metastasize to other organs, are less likely to be eliminated by the immune system and may have a higher survival rate in circulation. Therefore, identifying novel target genes and elucidating the mechanisms by which they endow malignant tumor cells with metastatic potential are vital for developing novel therapeutics to suppress metastasis and improve the prognosis of cancer patients.

In this study, we used a coculture model to explore platelets-mediated impacts on the metastatic potential of cancer cells and found that cocultured cancer cells displayed stronger survival and metastatic capacities than monocultured cells. By analysing the RNA sequencing results of these cocultured cells, we identified that serpin family E member 1 (SERPINE1) was highly upregulated after coculture. It belongs to the serpin E family with three members: SERPINE1, SERPINE2 and SERPINE3 41. SERPINE1 encodes plasminogen activator inhibitor 1 (PAI-1), which acts as a blood coagulation regulatory protein that can inhibit tissue plasminogen activator (tPA) and urokinase plasminogen activator (uPA), thus inhibiting fibrinolysis 42, 43. Previous research has mostly concentrated on the function of PAI-1 in thrombogenesis, while recent research has shown that a high PAI-1 concentration is an adverse prognostic factor in some types of cancers, such as lung 44, breast 45, gastric 46 and colorectal cancers 47. In this study, we discovered new functions of PAI-1 and elucidated the underlying mechanisms through which a high level of PAI-1 enabled cocultured cancer cells to display stronger capacities for tumorigenesis and metastasis.

## Materials and Methods

### Cell lines and cell culture

Melanoma B16F10 cells, TNBC MDA-MB-231 cells, and human embryonic kidney 293T cells were purchased from the American Type Culture Collection. They were cultured in Dulbecco's modified Eagle's medium (DMEM; #12100046, Thermo Fisher Scientific, USA), which contained 1% penicillin-streptomycin antibiotics (#15140122, Thermo Fisher Scientific, USA) and 10% fetal bovine serum (FBS; #10270-106, Gibco, USA). A FRET-based sensor C3 and green fluorescent protein (GFP) were transfected into B16F10 and MDA-MB-231 cells to generate B16F10-C3 and 231-GFP cells, respectively. B16F10-C3 cells emit green fluorescence, indicating that they are alive, and emit blue fluorescence when sensor C3 is cleaved by caspase-3/7 during apoptosis.

### Platelets isolation and purification

The average number of platelets in human blood is 1 to 3 × 10^8^ platelets per ml. Platelets were isolated using the following protocol which was optimized based on previously described method to acquire the maximum amount of platelets 48. Blood was drawn from mice through a cardiac puncture and collected in a test tube containing 150 µL of acid-citrate dextrose (ACD) buffer containing 39 mM citric acid, 75 mM sodium citrate, 135 mM dextrose, pH 7.4. The blood sample was centrifuged at 200 × g for 10 min at room temperature (RT). After centrifugation, the platelet-rich plasma (PRP) on the top layer was transferred to a new tube without disturbing the middle layer of the buffy coat. The PRP was mixed with an equal volume of wash buffer containing 10 mM sodium citrate, 150 mM NaCl, 1 mM EDTA, 1% (w/v) dextrose, pH 7.4, plus 1 μM prostaglandin E1 (PGE1, #P5515, Sigma-Aldrich, German). After several washes, the platelet pellets were collected at 1000 × g for 10 min. The platelets were resuspended in Tyrode's buffer containing 134 mM NaCl, 12 mM NaHCO_3_, 2.9 mM KCl, 0.34 mM Na_2_HPO_4_, 1 mM MgCl_2_, 10 mM HEPES, pH 7.4, and their number was counted using a haemocytometer. The platelets were kept in a 37°C CO_2_ incubator for 30 min before being added to cancer cells for the coculture experiments. The purity of mouse platelets is about 99% (Fig. S1D), for human platelets the purity should also be high.

### Establishment of a coculture model

After acquiring a specified number of platelets, we immediately mixed B16F10-C3 cells with mouse platelets at the ratios of 1:100 and 1:1,000 (cancer cells to platelets) and allowed them to circulate using the microfluidic circulatory pump to determine the protective effects of platelets. We used shear stress SS15 (15 dyne/cm^2^), which represents the average level of shear stress in human arteries at a resting status. Additionally, we used the same ratio and simultaneously cocultured cells for different time points, such as 3 h to 48 h. After the coculture, platelets were washed away to the maximum extent to exclude the influence of residual platelets on the experimental results. The cancer cells with remaining platelets were then allowed to circulate in the microfluidic circulatory system to determine cell survival ability. Finally, we found an optimized condition based on cell viability after circulatory treatment and used this condition for subsequent functional studies. Five to 10 ng/ml recombinant TGFβ1 protein (#7666-MB-005, R&D Systems, USA) and 2.5 μM SB431542 (#S1067, Selleckchem, USA) were added to the culture medium to treat the cancer cells for 24 h.

### Functional assays *in vitro*

Transwell chambers (#3422, Corning, USA) were used to perform the cell migration assay. One hundred thousand melanoma cells and ten thousand breast cancer cells were resuspended in 100 μL medium containing 2% FBS and seeded on the top layer, and 600 μL medium containing 10% FBS was added on the bottom. Eighteen hours later, the non-migrated cells in the upper layer were removed, and the migrated cells in the lower layer were fixed with 4% paraformaldehyde (PFA; #158127, Sigma-Aldrich, Germany) and stained with crystal violet (#C6158, Sigma-Aldrich, Germany). The whole field of the membrane was photographed under a microscope, and blue-stained cells were quantified.

An invasion assay was performed using the same protocol except the chambers were precoated with Growth Factor Reduced Matrigel. A colony formation assay was performed by seeding 1,000 cells in each well of 6-well plates and cultured for approximately 8 days for B16F10-C3 cells and 12 days for 231-GFP cells. At the end of the experiment, the cells were washed, fixed and stained. Colonies containing 50 or more cells were counted with ImageJ.

### MTT and cell proliferation assays

After circulatory treatment, 100 μL of circulated cells was collected and seeded in 96-well plates with six replicates. Ten microlitres of MTT (#M2128, Sigma-Aldrich, Germany) solution was added at a final concentration of 0.5 mg/ml. Four hours later, 10% SDS with 0.01 M HCl (100 μL) was added to each wall to dissolve the crystal product overnight. The absorbance at 595 nm was measured by a plate reader (PerkinElmer VICTOR X3, USA).

For measuring cell proliferation, 100 µL culture medium containing 2000 cells was added into a 96-well plate and incubated for 1 to 4 days. The cell growth rate was calculated based on the absorbance from the MTT assays.

### TGF-β1 enzyme-linked immunosorbent assay (ELISA)

To obtain conditioned medium from activated platelets, platelets were pretreated with thrombin (final concentration, 0.5 U/ml) at 37°C for 15 min. After centrifugation, the pellet containing the platelets was discarded and the supernatant was collected for the ELISA. For the none-activated platelets group, the freshly isolated platelets were incubated at 37°C for 24 h and the condition medium was collected after centrifugation. For 100 μL of culture media after different treatments, 20 μL of 1 N HCI were added to activate the latent form of TGF-β1 and the levels of TGF-β1 were measured using the DuoSet TGF-β1 ELISA kit (#DY1679-05, R&D Systems, USA).

### Metastasis assays *in vivo*

C57BL/6J or nude mice at 6 to 8 weeks of age were provided by the Animal Facility of the University of Macau and randomly divided into different groups. For lung metastasis assays, B16F10-C3 or 231-GFP cells were cocultured with homologous platelets at a ratio of 1:1,000 for 24 h. The cells were washed, trypsinized and counted; 2.5 × 10^5^ B16F10-C3 cells and 5.0 × 10^5^ 231-GFP cells were injected into the tail vein of C57BL/6J or nude mice, respectively. Twenty one or twenty eight days post injection, mice were sacrificed and the number of lung metastatic foci were determined based on the fluorescence signal by fluorescence microscopy.

For the orthotopic tumor model, cells were washed, trypsinized and counted. Fifty thousand B16F10-C3 cells were subcutaneously inoculated into C57BL/6J mice, and two million 231-GFP cells were injected into the mammary fat pads of the 4^th^ pair of nude mice. Mouse weight and tumor volume were monitored weekly.

### Haematoxylin and eosin (H&E) staining

After the mice were euthanized, lung tissues were dissected, fixed, and embedded in paraffin. A microtone (Leica Biosystems) was used to obtain a consecutive slice of each lung tissue, and the slices were stained with H&E (Leica Biosystems). Finally, photos of the stained slices were captured by the Aperio Scanner System (Leica).

### RNA extraction and qRT-PCR

The experiments were performed by following the protocol described previously 3. The primers for qRT-PCR are listed in Table S1. The concentration of RNA from platelets (equal in number to treated cells) was too low to perform reverse transcription reactions, so the RNA was not used for qRT-PCR.

### siRNA transfection and lentivirus infection

Different siRNAs targeting TGFβ1, TGFBR1, TGFBR2, Smad2, Smad3, and Smad4 were designed and purchased from General Biologicals. Lipofectamine 3000 was purchased from Invitrogen. siRNA was diluted using Opti-MEM and mixed with diluted Lipofectamine (1:1 ratio) for 10 to 15 min. The cells were incubated with siRNA complexes for 48 h, and knockdown efficiency was evaluated by qRT-PCR. Detailed information on the siRNA sequences is shown in Table S2. The PAI-1 shRNAs or overexpression vectors are listed in Table S2 and Table S3. The packaging plasmids, dR8.2, and targeting plasmid were mixed at a weight ratio of 1:2:3. Lentiviruses were collected after the mixture was transfected into 293T cells for 48 to 72 h. Cancer cells were infected and selected to generate stable cells with target gene being knocked down or overexpressed.

### Western blot analysis

The experiments were performed by following the protocol described previously 3. Primary antibodies against TGFβ1 (ab215715), TGFBR1 (ab31013), TGFBR2 (ab1868383), PAI-1 (ab222754), SERPINE2 (ab154591), p-Bad (ab28824), and Bad (ab32445) were obtained from Abcam (1:1000). Primary antibodies against p-Smad2/3 (#8828), Smad2/3 Antibody Sampler Kit (#12747), p-PI3K (#4228), PI3K (#4249), p-AKT473 (#4060), p-AKT308 (#13038), AKT (#4685), Bcl-2 (#2870), and GAPDH (#2118) were purchased from Cell Signaling Technology (diluted at 1:1000). Primary antibodies against p-TGFBR1 (PA5-40298) were obtained from Thermo Fisher.

### Immunofluorescence (IF) staining

Cancer cells were seeded onto coverslips one day before coculture. After coculturing, IF staining was performed as previously described 3. Finally, the fluorescent images of PAI-1 stained cells were captured by a confocal fluorescence microscope (Carl Zeiss Confocal LSM710, Germany).

### Immunohistochemistry (IHC)

The catalogue numbers for the tissue microarray (TMA) slides were HMelC112CD01 for melanoma and F551101 for TNBC. Tumor samples were analysed using the IHC Detection Kit (ab64264, Abcam). AEC was used as a chromogen for melanoma samples, and diaminobenzidine was used for TNBC samples by strictly following the indicated protocols. Images were captured by the Aperio Scanner System. PAI-1 expression was scored manually based on staining intensity, ranging from 0 to 3 at an interval of 0.5. PAI-1 staining in each sample was scored as high (score ≥ 2), medium (1 ≤ score < 2) or low (score < 1) staining, and samples were classified as positive (score ≥ 1.5) or negative (score < 1.5) based on this score.

### Statistical analysis

Statistical results were analysed by Prism software (GraphPad Software Inc., USA) and presented as the mean ± SD or SEM as indicated. Experiments were performed at least three times. One- or two-way ANOVA, chi-square test and Student's t test were used to determine the statistical significance; *P < 0.05, **P < 0.01, ***P < 0.001 and ns indicates no significance.

## Results

### Shear stress could kill B16F10-C3 cells in a microfluidic circulatory system, while coculture with platelets greatly increased cell survival in circulation

**Figure 1:**
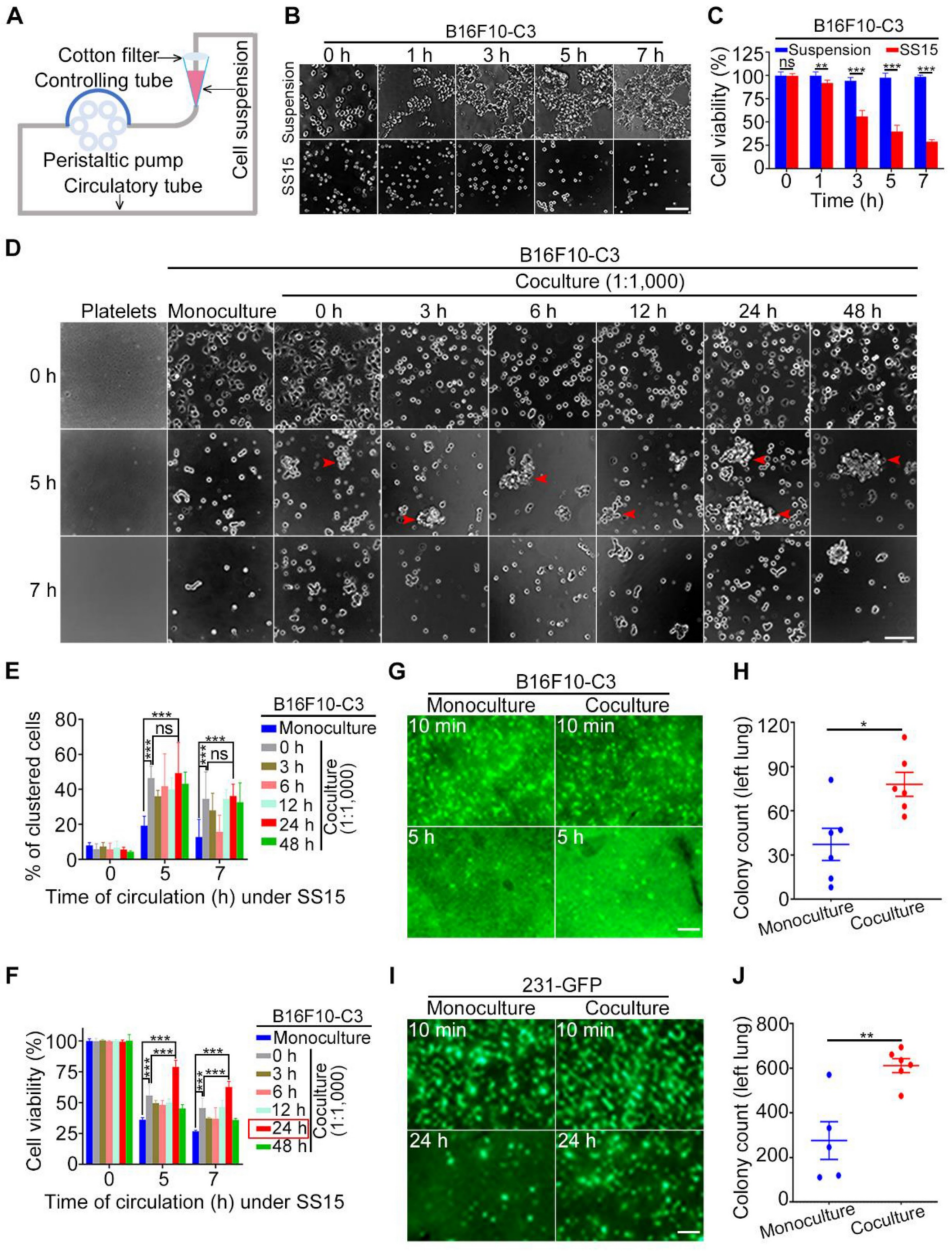
Coculture with platelets protected cancer cells against SS damage *in vitro* and *in vivo*. (**A**) The design of a microfluidic circulatory pump. (**B** and **C**) Representative images and quantification of B16F10-C3 cell viability after SS15 or suspension treatment. Scale bar, 100 μm. (**D**) Phase images of murine platelets, monocultured and cocultured B16F10-C3 cells (cocultured with murine platelets at 1:1,000 for different time periods) after 0, 5 and 7 h of SS treatment. The red arrowheads indicate clustered cells. Scale bar, 100 μm. (**E** and **F**) Quantified % of clustered cells and cell viability of B16F10-C3 cells after SS treatment. (**G** and **H**) Representative images and quantification of GFP^+^ foci in the lung 5 h after tail vein injection of monocultured and cocultured B16F10-C3 cells in mice (n = 6). (**I** and **J**) Fluorescent images and quantification of GFP^+^ foci in the lung 24 h after tail vein injection of monocultured and cocultured 231-GFP cells in mice (n = 5 to 6). Scale bar, 1 mm. The data are shown as the mean ± SD. *P < 0.05, **P < 0.01, ***P < 0.001 and ns, not significant.

When blood flows through the vascular system in the human body, it can generate high levels of SS (ranging from 4-30 dyne/cm^2^) in arteries; it can also generate low levels of SS (ranging from 0.5-4 dyne/cm^2^) in veins 49, 50. Previously, we developed a peristaltic pump that can generate different levels of SS, which was used to study its effects on CTCs (Fig. 1A) 2, 3, 51. In this study, we used an SS of 15 dyne/cm^2^, whose level represents human arteries in the resting state. B16F10-C3 cells were trypsinized, and 2 × 10^5^ cells in one millilitre culture medium were allowed for circulatory treatment for 0 to 7 h. The same number of cells under the suspension condition for 0 to 7 h were regarded as control groups. The phase contrast images indicated that the number of cells was decreased after circulatory treatment (Fig. 1B). The quantified MTT results showed that cell viability was reduced by 60 to 75% after SS15 treatment for 5 to 7 h (Fig. 1C).

Next, we tested whether coculturing B16F10-C3 cells with C57BL/6J mouse-derived platelets at a 1:1,000 ratio for 0 to 48 h could affect cell viability. After circulation for 5 or 7 h, cells from four channels were mixed, and 100 µL of cell suspension was added into a 96-well plate with six replicates for each condition. After 5 min of cell sedimentation, phase contrast images and quantified results showed that the existence of platelets could significantly enhance the percentage of clustered cells between monoculture and coculture (Fig. 1D and E). Importantly, 24 h of coculture with platelets produced the highest increase in viability compared with the other 5 time points of coculture (0, 3, 6, 12, 48 h). Furthermore, at a 1:1,000 ratio between cancer cells and platelets, a 5 h circulation time resulted in higher viability than a 7 h circulation time (79% vs. 63%) (Fig. 1F). Finally, a coculture ratio of 1:100 had fewer (14%) protective effects on the viability of circulated cancer cells (Fig. S1A to C). Because coculturing tumor cells with platelets at a 1:1,000 ratio for 24 h produced the best protective effects against 5 h SS-induced cell death, we used this condition for the following animal experiments.

Next, we injected monocultured or cocultured B16F10-C3 cells into C57BL/6J mice through the tail vein and compared the *in vivo* survival ability by counting the number of GFP^+^ colonies 5 h after injection. Fluorescence microscopy analysis showed that the 24 h coculture treatment significantly increased the number of GFP^+^ colonies in the largest lung lobe from 37 to 78 (Fig. 1G, H). To confirm the above observation, we used another metastatic TNBC cell line, 231-GFP, to perform the same type of animal experiments. From our previous experience, we learned that for monocultured 231-C3 cells, 12 h of circulatory treatment only induced a small portion of circulated cells to undergo apoptosis 2. Therefore, in this study, we calculated the number of GFP^+^ colonies located in the lung 24 h after injecting the cells into the tail vein of mice.

Fluorescence images and quantified results showed that after coculturing 231-GFP cells with human platelets, the cocultured group displayed higher survival and homing abilities to the lung than the monoculture group. Specifically, the average colony count of the coculture group was significantly increased by 2.2-fold (from 276 to 612 colonies) (Fig. 1I, J). Together, these findings suggest that when 231-GFP or B16F10-C3 human- or mouse-derived cancer cells were cocultured with species-homologous platelets, these cocultured cancer cells displayed stronger survival ability in circulation and lung colony formation ability *in vivo* than monocultured cancer cells.

### Coculture with platelets enhanced the migration, invasion, and colony formation of cancer cells and promoted tumorigenesis and metastasis

**Figure 2:**
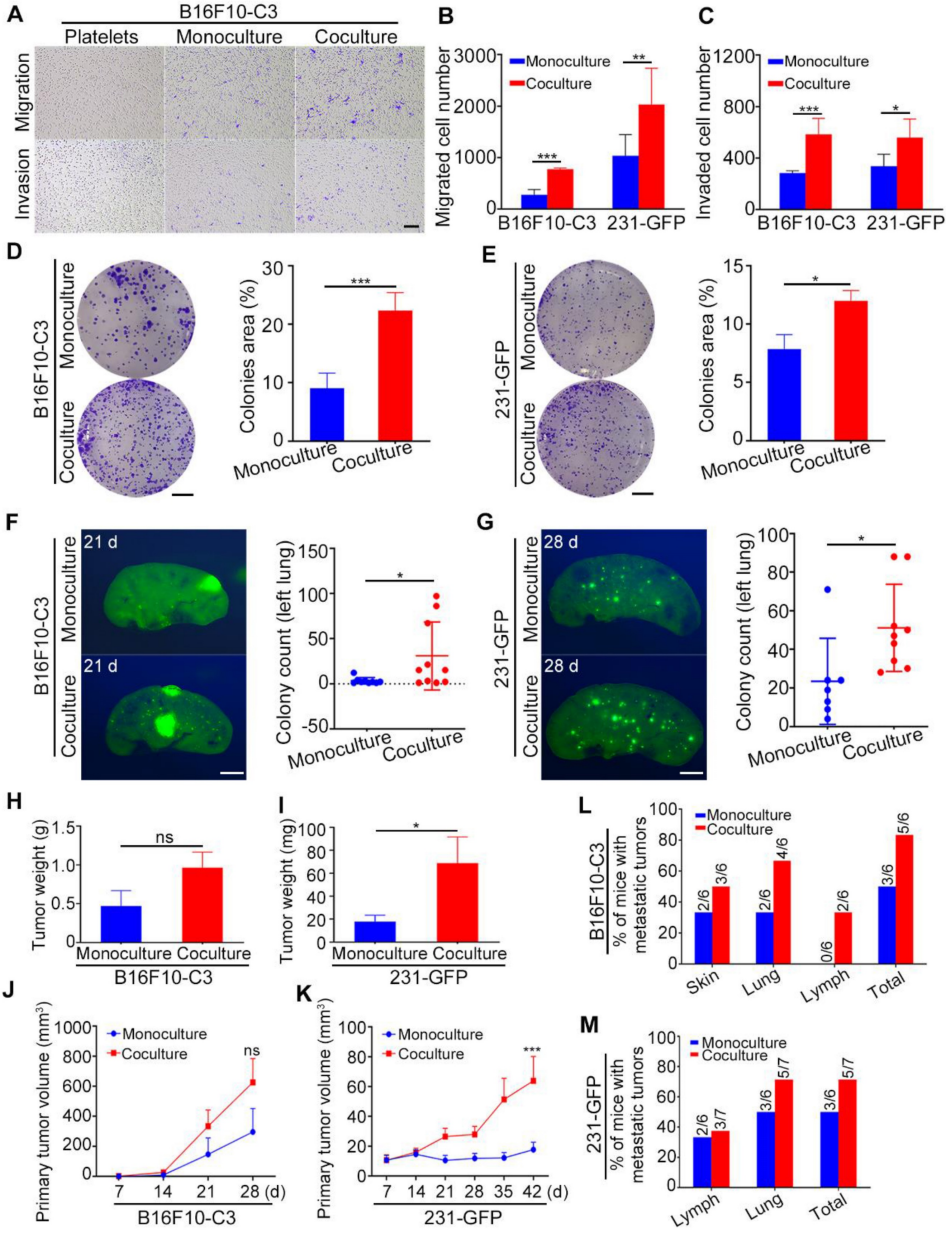
Cocultured cancer cells showed increased migration, invasion, colony formation, tumorigenic and metastatic capacities. (**A**) Transwell migration and invasion assays for platelets and monocultured and cocultured B16F10-C3 cells. Scale bar, 200 μm. (**B** and **C**) Quantification of the migrated and invaded B16F10-C3 and 231-GFP cells in the monocultured and cocultured groups. (**D** and **E**) Images and quantification of the colony formation assays for the monocultured and cocultured B16F10-C3 and 231-GFP cells. Scale bar, 5 mm. (**F** and **G**) Two hundred and fifty thousand B16F10-C3 cells or half a million 231-GFP cells in the monocultured and cocultured groups were tail-vein injected into C57BL/6J (n = 9 to 10) and nude mice (n = 7 to 9), respectively. Mice were sacrificed 21 days or 28 days post injection. Colonies formed in the left lungs were counted, and representative images are shown. Scale bar, 1 mm. (**H** and **I**) The primary tumor weights in the monocultured and cocultured B16F10-C3 and 231-GFP cells using the orthotopic models. (**J** and **K**) The quantified results for primary tumor volume. (**L** and **M**) Quantification % of mice with metastatic tumors. The results are shown as the mean ± SD, except for the data of tumor weight and tumor volumes, which are mean ± SEM. *P < 0.05, **P < 0.01, ***P < 0.001 and ns indicates no significance.

Next, we conducted a series of functional studies to determine what phenotypes were changed in cancer cells after coculture with homologous platelets. The migration and invasion abilities of B16F10-C3 and 231-GFP cells were significantly increased (approximately 2-fold) after coculture (Fig. 2A to C, Fig. S2A), while cell proliferation was not affected (Fig. S2B and C). In addition, more colonies were formed by the cocultured cells than by the monocultured cells, with increases of 2.4- and 1.5-fold for B16F10-C3 and 231-GFP cells, respectively (Fig. 2D and E). Three or four weeks post tail vein injection, the number of GFP^+^ foci in the left lungs of mice was significantly increased in the coculture groups compared with the monoculture groups (Fig. 2F and G, Fig. S2D and E).

To further investigate the tumorigenic and metastatic capacities of cocultured cells in orthotopic models, we injected monocultured and cocultured B16F10-C3 cells subcutaneously into C57BL/6J mice and two groups of 231-GFP cells into the fat pads of nude mice (Fig. S2F and G). The body weights of the mice were measured weekly, and there was no difference between the monocultured and cocultured cells (Fig. S2H and I). Twenty-eight days post injection of B16F10-C3 cells and 42 days post injection of 231-GFP cells, the primary tumors were dissected and weighed after mice were sacrificed. In terms of B16F10-C3 cells, primary tumors were formed by monocultures in five of the six mice, while cocultured cells could form primary tumors in all six mice (Fig. S2J). In terms of 231-GFP cells, the primary tumor formation rate was 58.3% (7/12) in the monoculture group and 62.3% (10/14) in the coculture group (Fig. S2K). The tumors formed by cocultured 231-GFP cells were larger and heavier than those formed by monocultured cells, while no significant changes were found in B16F10-C3 cells after coculture (Fig. 2H-K, Fig. S2F and G). These results showed that coculture with platelets enhanced the tumorigenesis of 231-GFP cells but not B16F10-C3 cells.

For metastasis of B16F10-C3, only three of the six mice formed tumors that metastasized to the distant skin and lung in the monoculture group, while five of the six mice in the skin, lung, and iliac lymph node had metastatic tumors in the coculture group (Fig. 2L, Fig. S2L). Similarly, cocultured 231-GFP cells showed higher metastatic capacity than monocultured cells. Metastasis in the iliac lymph node and lung occurred in five of the seven mice in the coculture group, while only three of the six mice in the monoculture group had metastatic tumors (Fig. 2M, Fig. S2M). The lymphatic metastasis area was not significantly increased in the 231-GFP cell group (Fig. S2N). Taken together, these results indicate that the interaction between tumor cells and platelets increased the metastatic potential of both types of tumor cells and only enhanced the tumorigenic capacity of 231-GFP cells.

### SERPINE1 was identified as an important upregulated gene in cocultured tumor cells

**Figure 3:**
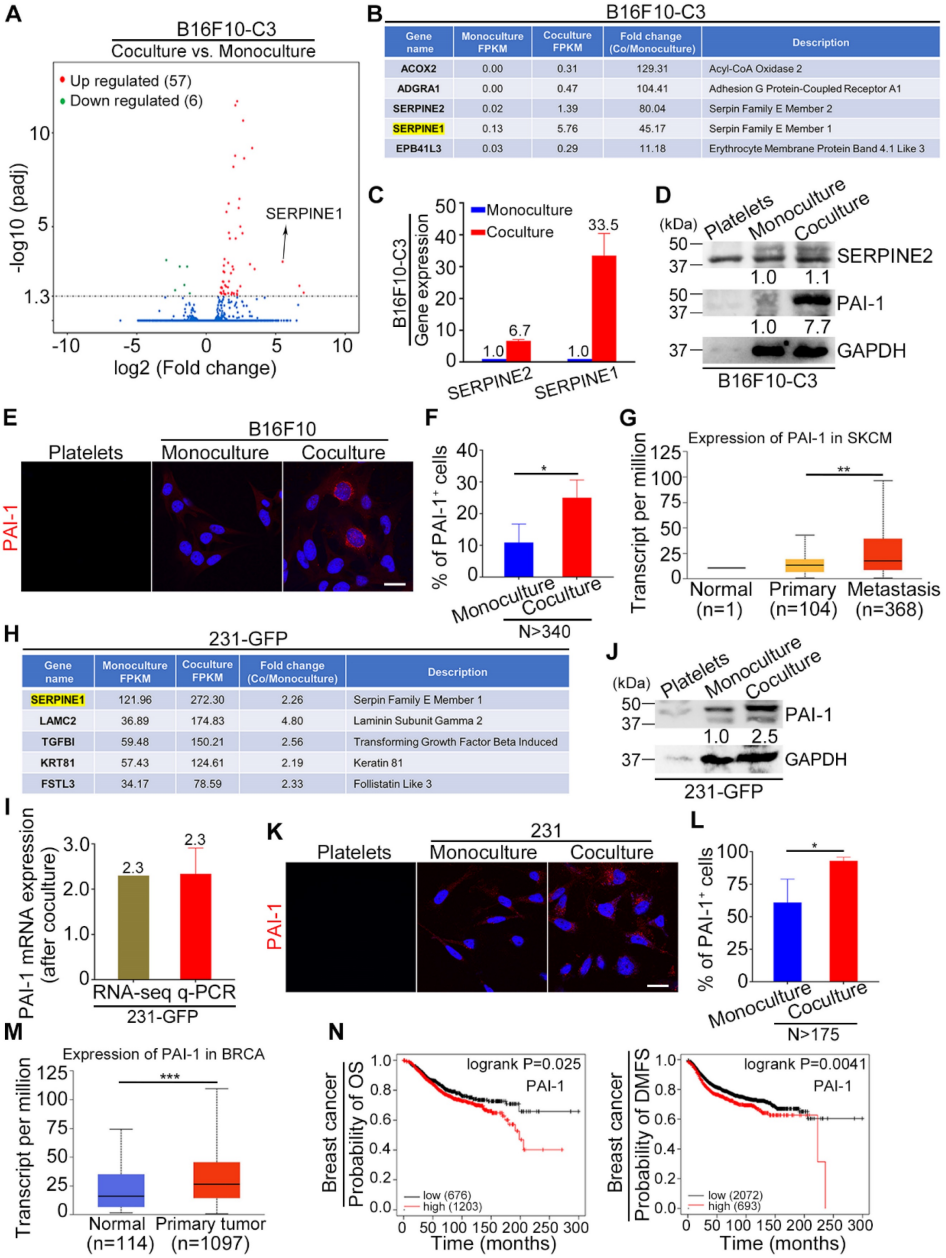
PAI-1 was significantly upregulated in cocultured B16F10-C3 and 231-GFP cells. (**A**) Volcano plot showing the gene profiles in cocultured vs. monocultured B16F10-C3 cells. Red and green dots indicate the upregulated and downregulated genes, with significant increases of more than 2-fold, respectively. (**B**) List of the top 5 upregulated genes in cocultured B16F10-C3 cells. (**C**) Validation of SERPINE2 and SERPINE1 mRNA levels by qRT-PCR. (**D**) Western blotting shows the SERPINE2 and PAI-1 protein levels in murine platelets, monocultured and cocultured B16F10-C3 cells. Equal quantities of platelets used for coculture were loaded as a control. (**E**) Representative IF images of PAI-1 distribution in murine platelets and monocultured and cocultured B16F10 cells. Scale bar, 20 μm. (**F**) Quantified results of PAI-1-positive (PAI-1^+^) cells in monocultured and cocultured B16F10 cells (N > 340). (**G**) Correlation of PAI-1 expression in normal, primary and metastatic tissues in patients with skin cutaneous melanoma from the TCGA database. (**H**) List of the upregulated genes with the top 5 highest FPKM values in cocultured 231-GFP cells. (**I** and **J**) Validation of PAI-1 expression by qRT-PCR and Western blotting. (**K**) Representative IF images of PAI-1 distribution in human platelets, monocultured and cocultured 231 cells. Scale bar, 20 μm. (**L**) Quantified results of PAI-1^+^ cells in monocultured and cocultured 231 cells (N > 175). (**M**) The correlation of PAI-1 expression between normal and primary tumors in breast invasive carcinoma patients from the TCGA database. (**N**) Kaplan-Meier plots showing the correlation of PAI-1 with overall survival (OS) and distant metastasis-free survival (DMFS). The results are shown as the mean ± SD. *P < 0.05, **P < 0.01, ***P < 0.001.

To identify key genes that are responsible for the increased tumorigenesis and metastasis of tumor cells after coculture with platelets, we conducted RNA sequencing (RNA-seq) analysis of monocultured and cocultured B16F10-C3 cells. The RNA-seq results showed that 57 genes were upregulated and 6 genes were downregulated after coculture with statistical significance (Fig. 3A). Among the top 5 upregulated genes, SERPINE1 had the highest fragments per kilobase of transcript per million mapped reads (FPKM) value in both the monocultured and cocultured cells, with a fold change of 45.17-fold after coculturing, making it easier to detect and further investigate (Fig. 3B). We compared the expression levels of two members of the serpin E family, SERPINE1 and SERPINE2, and found that the mRNA level of SERPINE1 was increased 33.5-fold, while the mRNA level of SERPINE2 only increased 6.7-fold after coculture (Fig. 3C). At the protein level, only PAI-1 (encoded by SERPINE1) was elevated 7.7-fold in the cocultured B16F10-C3 cells (Fig. 3D). We then evaluated the location of PAI-1 by IF staining, and the fluorescent images showed obvious enhancement of PAI-1 near the nuclear region in B16F10 cells after coculture (Fig. 3E). The percentage of PAI-1-positive (PAI-1^+^) cells was also significantly increased from 11% to 25% after coculture (Fig. 3F). In addition, these results revealed the heterogeneous expression pattern of PAI-1 in B16F10 cells. From the TCGA database, we found that the expression of PAI-1 was much higher in the metastasis group than in the primary group of skin cutaneous melanoma (SKCM) patients (Fig. 3G).

In the RNA-seq results of 231-GFP cells, we found that SERPINE1 was also significantly increased after coculture and had the highest FPKM value in both the monocultured and cocultured groups, suggesting its importance during the interactions between cancer cells and platelets (Fig. 3H). The upregulation of SERPINE1 was well validated by qRT-PCR and Western blotting, which showed 2.3-2.5-fold increases at the mRNA and protein levels, respectively (Fig. 3I, J). Moreover, the IF staining assays revealed that the percentage of PAI-1-positive cells was enhanced by 1.5-fold after coculture (Fig. 3K, L). The heterogeneous expression pattern of PAI-1 in 231 cells was also observed. According to the TCGA database, the expression of PAI-1 was significantly higher in primary tumors than in normal tissues from breast cancer patients (Fig. 3M). Kaplan-Meier plots displayed that high expression of PAI-1 was correlated with shorter overall survival (OS) and distant metastasis-free survival (DMFS) in breast carcinoma patients (Fig. 3N). Overall, SERPINE1 was identified as an important platelets-stimulated gene based on its upregulation in cocultured tumor cells.

### Knocking down PAI-1 attenuated the promoting effects of platelets during coculture on cancer cell survival in circulation, tumorigenesis and metastasis

**Figure 4:**
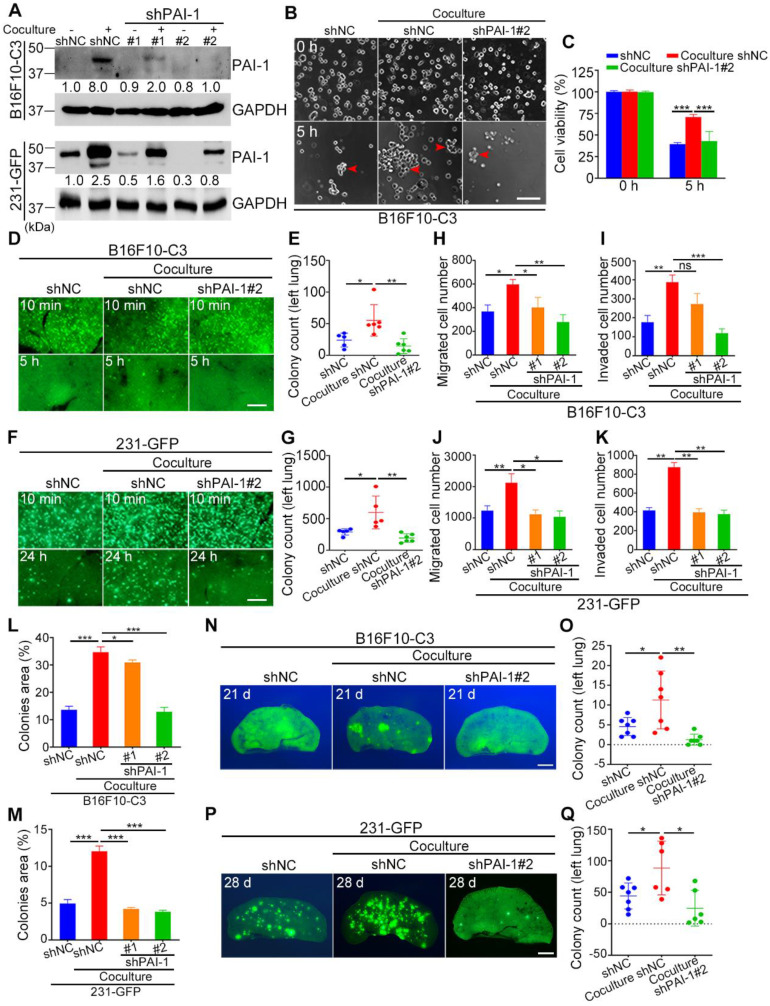
Knockdown of PAI-1 effectively reduced the survival and metastatic potential of B16F10-C3 and 231-GFP cells. (**A**) Western blotting results showing the protein levels of PAI-1 after knocking down PAI-1 with shRNAs. (**B** and **C**) Representative phase contrast images and quantified results of cell viability of B16F10-C3 cells for 5 h under SS15 after knockdown of PAI-1. Scale bar, 100 μm. (**D** to **G**) Fluorescent images and quantification of cell survival in circulation *in vivo* after knocking down PAI-1 in B16F10-C3 and 231-GFP cells 5 h and 24 h post injection through the tail vein in C57BL/6J (n = 5 to 6) and nude mice (n = 5 to 6), respectively. Scale bar, 1 mm. (**H** to **K**) Quantified results of the Transwell migration and invasion assays for monocultured shNC, cocultured shNC, cocultured shPAI-1#1 and shPAI-1#2 B16F10-C3 and 231-GFP cells. (**L** and **M**) Quantified results of the colony formation assay for monocultured shNC, cocultured shNC, cocultured shPAI-1#1 and shPAI-1#2 B16F10-C3 and 231-GFP cells. (**N** to **Q**) Fluorescent images and colony count after knocking down PAI-1 in B16F10-C3 cells at 21 days in C57BL/6J mice (n = 7) and 231-GFP cells at 28 days in nude mice (n = 6 to 7) post injection through the tail vein, respectively. Scale bar, 1 mm. The results are shown as the mean ± SD. *P < 0.05, **P < 0.01, ***P < 0.001 and ns, no significance.

To explore the potential role of PAI-1 in cancer progression promoted by platelets, we used two shRNAs to reduce the levels of PAI-1. The knockdown cells were cocultured with platelets and used in a series of experiments. The efficiency of shRNA knockdown was validated using qRT-PCR and Western blotting, and shPAI-1#2 had a better knockdown effect than shPAI-1#1, so it was selected for circulatory and *in vivo* experiments (Fig. S3A, B and Fig. 4A). To determine the impact of knockdown of PAI-1 on cell survival, B16F10-C3 cells with shPAI-1#2 were cocultured with platelets and allowed to circulate in the microfluidic circulation pump under SS15 for 5 h. These cells formed fewer clusters and had a lower survival rate in circulation than the cocultured shNC cells. The quantified results showed that cell viability decreased from 71% to 43% after knockdown of PAI-1 (Fig. 4B, C).

To investigate whether knockdown of PAI-1 also affected cell survival in circulation *in vivo*, three groups of B16F10-C3 cells (monocultured shNC, cocultured shNC and cocultured shPAI-1#2 cells) were inoculated into C57BL/6J mice through the tail vein. At 5 h post injection, mice were sacrificed, and the number of GFP^+^ colonies in the lung was counted. The results indicated that the number of colonies formed by B16F10-C3 cells in the lung significantly decreased by over 2.5-fold after PAI-1 knockdown (Fig. 4D, E). In 231-GFP cells, knockdown of PAI-1 also led to a significant reduction in lung colonies 24 h post injection in nude mice. The average number decreased by over 3-fold (from 598 to 193 colonies) (Fig. 4F, G). In summary, decreasing the expression of PAI-1 reduced cell survival in circulation both *in vitro* and *in vivo*.

Next, we verified the function of PAI-1 in cell migration and invasion. The results of Transwell migration and invasion assays showed that knockdown of PAI-1 decreased the capacities of both B16F10-C3 and 231-GFP cells even when they interacted with platelets (Fig. 4H-K, Fig. S3C, D). The colony formation abilities of cocultured tumor cells were also decreased after knockdown of PAI-1 (Fig. 4L, M, Fig. S3E, F). In most of the aforementioned phenotypes, the knockdown effects were more obvious in shPAI-1#2 cells, which was consistent with the better knockdown efficiency of shPAI-1#2 vs. shPAI-1#1. The experimental pulmonary metastatic model demonstrated that the lung colony formation abilities of cocultured B16F10-C3 and 231-GFP cells were significantly attenuated due to reduced levels of PAI-1, illustrated by dramatic 88.6% and 71.9% decreases in colony number compared with that in the control groups for both types of malignant cancer cells (Fig. 4N-Q, Fig. S3G, H).

**Figure 5:**
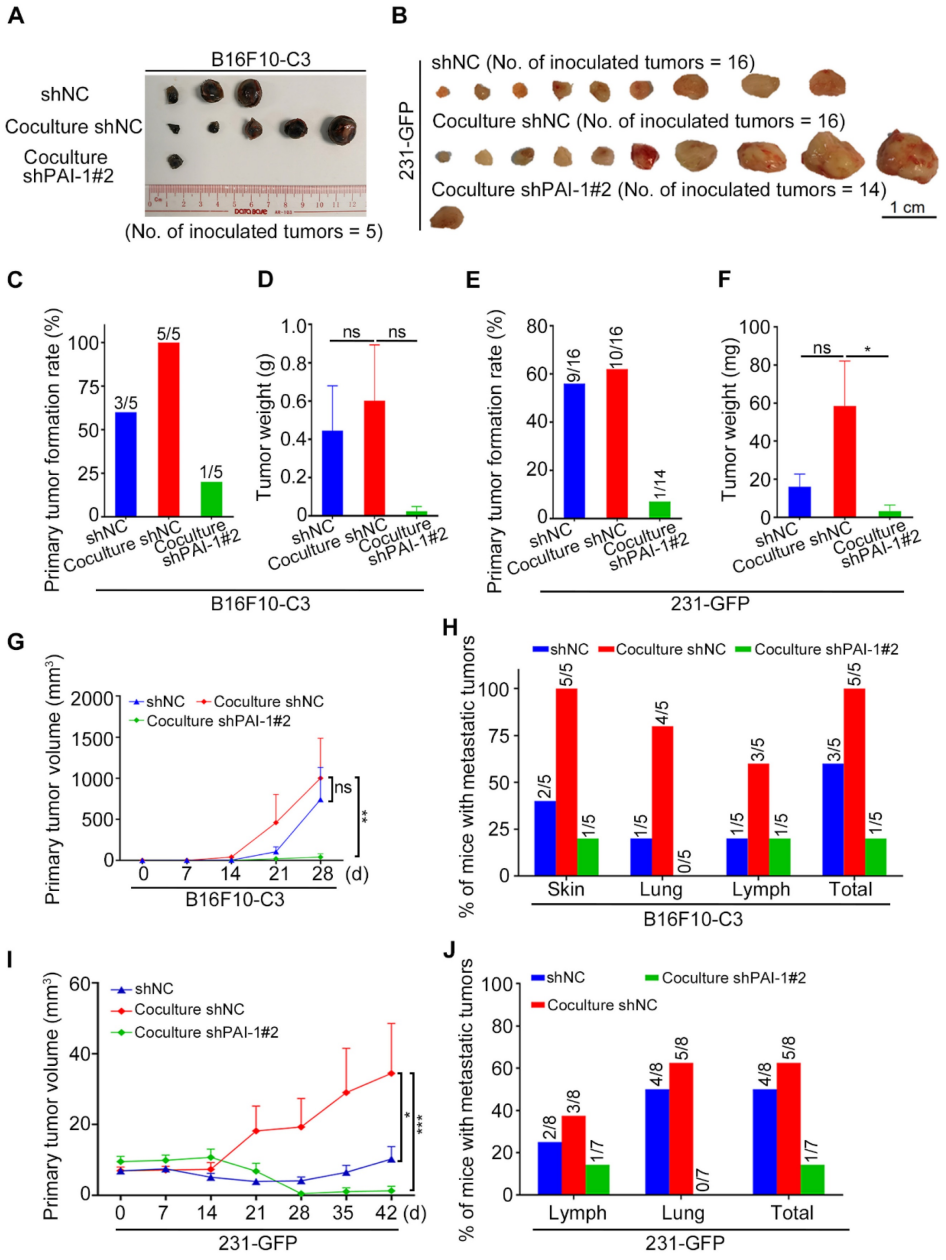
Knockdown of PAI-1 effectively suppressed primary tumor growth and metastasis. (**A** and **B**) Representative images of the primary tumors of monocultured shNC, cocultured shNC and cocultured shPAI-1#2 B16F10-C3 and 231-GFP cells, which were inoculated into C57BL/6J mice subcutaneously (No. of mice = 5) and into nude mice through fat pads (No. of mice = 7 to 8). (**C** to **F**) Quantified results of the primary tumor formation rate and tumor weight after knockdown of PAI-1 in B16F10-C3 and 231-GFP cells. (**G** and **I**) Quantified results of the primary tumor volume after knockdown of PAI-1 in B16F10-C3 and 231-GFP cells. (**H** and **J**) Quantified % of mice with metastatic tumors in B16F10-C3 and 231-GFP cells, respectively. The quantification is the mean ± SEM. *P < 0.05, **P < 0.01, ***P < 0.001 and ns, not significant.

In orthotopic models, monocultured B16F10-C3-shNC cells and cocultured shNC and shPAI-1#2 cells were injected into C57BL/6J mice, and the three groups of 231-GFP cells were injected into nude mice. Mouse weight was determined weekly, and the primary tumors were dissected after the mice were sacrificed (Fig. S3I and J, Fig. 5A and B). After knocking down PAI-1 in B16F10-C3 cells, the primary tumor weights were not significantly different, as only one tumor formed, but the tumor formation rate and tumor volumes were significantly decreased (Table S4, Fig. 5C, D, G).

For metastasis, only one of the five mice had metastatic tumors at a distant skin site and in the iliac lymph node in the knockdown group of PAI-1. In contrast, metastasis was observed in all five mice from the control group (Fig. 5H, Fig. S3K). In terms of 231-GFP cells, the primary tumor formation rate, tumor weight and tumor volume were all greatly decreased after knocking down PAI-1 (Fig. 5E, F, I, Table S5). In addition, only one of the seven mice had metastatic tumors in the iliac lymph node, while five of the eight mice had metastasis in the iliac lymph node and lung in the control group (Fig. S3L, M, Fig. 5J). These findings proposed that knocking down PAI-1 attenuates the promoting effects of platelets during coculture on cancer cell survival in circulation, tumorigenesis and metastasis for B16F10-C3 and 231-GFP cells.

### Overexpression of PAI-1 in B16F10-C3 cells enhanced cell survival in circulation, tumorigenesis and metastasis

**Figure 6:**
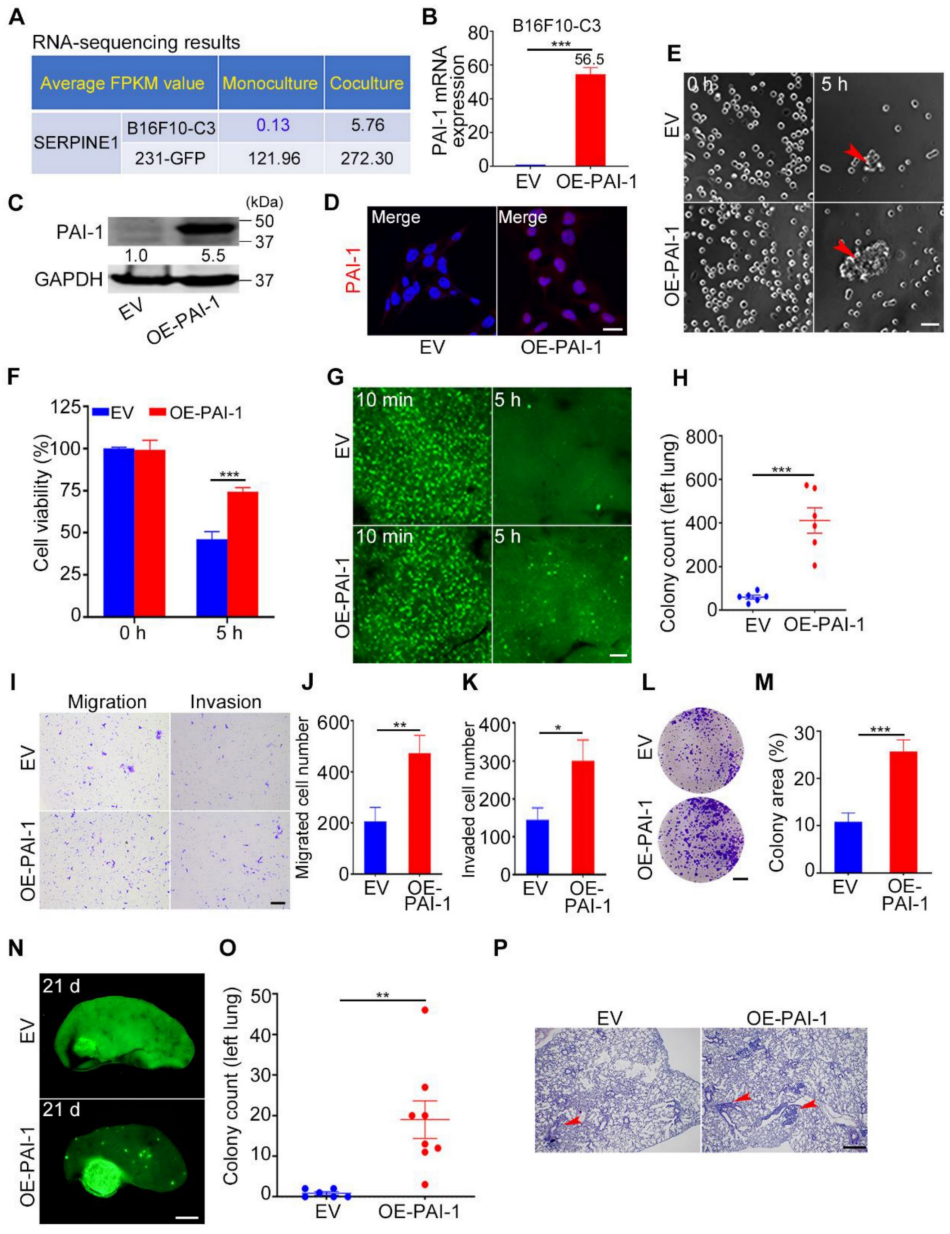
Overexpression of PAI-1 in B16F10-C3 cells enhanced cell survival and metastasis. (**A**) Average FPKM value of SERPINE1 encoding PAI-1 in monocultured and cocultured B16F10-C3 and 231-GFP cells. (**B** and **C**) The mRNA and protein levels of PAI-1 in the EV and OE-PAI-1 groups. (**D**) Representative IF images of PAI-1 distribution. Scale bar, 20 μm. (**E** and **F**) Representative contrast images and quantification of cell viability between EV and OE-PAI-1 cells. Scale bar, 100 μm. (**G** and **H**) Fluorescent images and colony count in left lung 5 h post tail vein injection of EV and OE-PAI-1 cells (n = 6). Scale bar, 1 mm. (**I** to **K**) Images and number of migrated and invaded EV and OE-PAI-1 cells. Scale bar, 200 μm. (**L** and **M**) Images and quantification of the colony formation assay for the EV and OE-PAI-1 cells. Scale bar, 5 mm. (**N** and **O**) Fluorescent images and colony count after overexpression of PAI-1 21 days post injection through the tail vein (n = 6 to 8). Scale bar, 1 mm. (**P**) H&E staining results of the mouse lung lobe. Scale bar, 20 μm. The results are displayed as the mean ± SD. *P < 0.05, **P < 0.01, ***P < 0.001.

To further validate the cancer-promoting role of PAI-1, we overexpressed PAI-1 in B16F10-C3 cells, in which the basal level of PAI-1 was much lower than that in 231-GFP cells according to RNA-seq results (Fig. 6A). The overexpression efficiency was well validated by qRT-PCR, Western blotting, and IF staining (Fig. 6B-D). In addition, overexpression of PAI-1 (OE-PAI-1) in B16F10-C3 cells significantly increased cell viability to 1.5-fold after SS15 treatment for 5 h in comparison to the empty vector (EV) group (Fig. 6E, F).

We further examined the cell survival ability by applying an experimental lung metastatic model. The results illustrated that higher PAI-1 expression greatly enhanced the capacity of B16F10-C3 cells to survive in circulation by nearly 7-fold (Fig. 6G, H). Then, we found that overexpression of PAI-1 effectively increased the migration, invasion, and colony formation abilities of B16F10-C3 cells (Fig. 6I-M). In addition, the lung metastatic ability of B16F10-C3 cells was significantly increased by overexpression of PAI-1, as the average colony count increased from 1 to 19 (Fig. 6N-P).

To examine the effects of PAI-1 overexpression on tumorigenesis and spontaneous metastasis, 5 × 10^4^ EV and OE-PAI-1 cancer cells were subcutaneously injected into C57BL/6J mice. The mouse body weight was measured weekly (Fig. S4D). The primary tumor formation rate in the overexpression group was 1.5-fold higher than that in the EV group. Although the average tumor weight was not significantly changed after overexpression, the tumor size was increased approximately 6-fold (Table S4, Fig. S4A-C, E). The metastasis formation rate was also higher in the overexpression group (Fig. S4F, G). This evidence illustrates that the overexpression of PAI-1 could promote cancer cell survival in circulation, tumorigenesis and metastasis, and these phenotypical changes have also been observed in cancer cells cocultured with platelets.

### Platelets promoted the survival and metastasis of malignant tumor cells by activating the TGFβ/Smad and PI3K/AKT pathways

**Figure 7:**
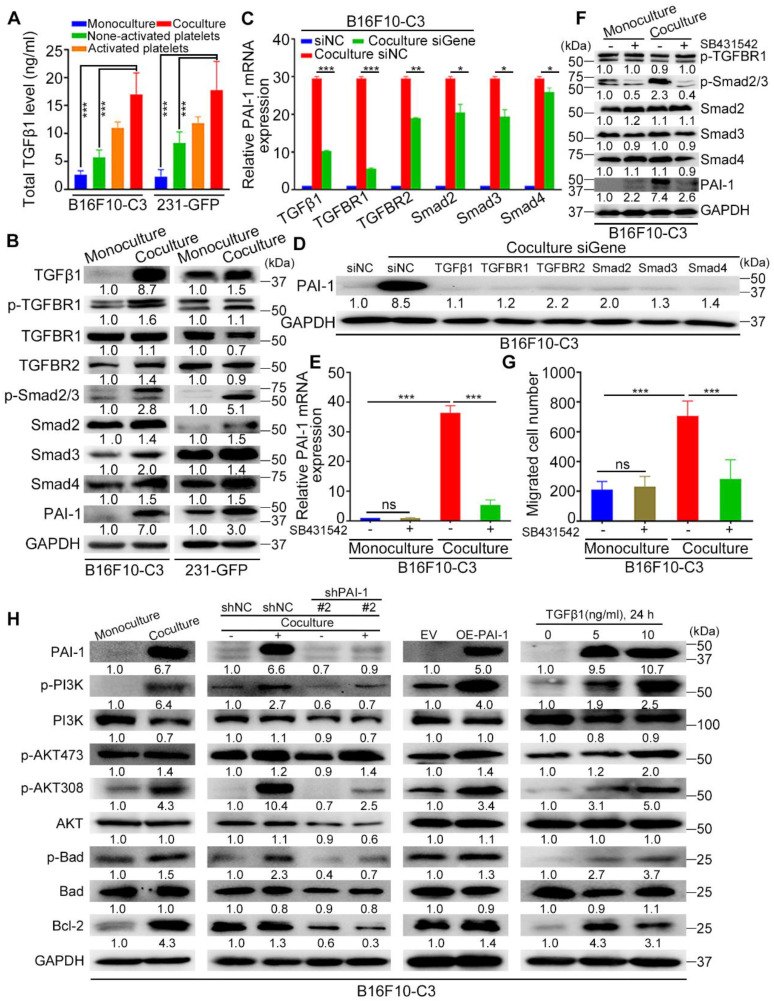
PAI-1-related signalling pathways were activated after coculture. (**A**) Total TGFβ1 level in the culture media of monocultured tumor cells, non-activated platelets, activated platelets, and B16F10-C3 and 231-GFP cells cocultured with non-activated platelets through ELISAs. (**B**) Western blotting shows the levels of TGFβ/Smad pathway-related proteins. (**C**) The mRNA levels of PAI-1 in B16F10-C3 cells after knocking down TGFβ1, TGFBR1, TGFBR2, Smad2, Smad3 and Smad4 using siRNAs. (**D**) PAI-1 protein levels after knocking down targeted genes. (**E**) mRNA levels of PAI-1 in B16F10-C3 cells after treatment with the TGFBR1 inhibitor SB431542. (**F**) The protein levels of p-TGFBR1, p-Smad2/3, Smad2-4, and PAI-1 after SB431542 treatment in B16F10-C3 cells. (**G**) Quantified results of the Transwell migration assay in B16F10-C3 cells after SB431542 treatment. (**H**) The protein levels of the PI3K/AKT pathway between monocultured and cocultured B16F10-C3 cells with knockdown and overexpression of PAI-1 and recombinant TGFβ1 protein treatment. The results are displayed as the mean ± SD. *P < 0.05, **P < 0.01, ***P < 0.001.

Platelets contain α-granules that are regulators of cell growth and angiogenesis, such as TGFβ, which can be released upon activation of platelets. Thus, we measured the concentration of TGFβ1 in the culture media of monocultured tumor cells, non-activated platelets, platelets activated by thrombin and B16F10-C3 and 231-GFP cells cocultured with non-activated platelets using ELISAs. The levels of secreted TGFβ1 in the coculture groups were increased approximately 6-fold compared to those in the monoculture groups in both B16F10-C3 and 231-GFP cells (Fig. 7A). We further found that the protein levels of TGFβ1 and its downstream proteins, including p-TGFBR1, p-Smad2/3, Smad2-4 and the TGFβ-responsive gene SERPINE1 (encodes PAI-1), were elevated after coculturing in cancer cells (Fig. 7B).

Next, we used gene-specific siRNAs to knockdown these target genes in B16F10-C3 cells, and the knockdown efficiencies were over 60% at both the mRNA and protein levels (Fig. S5A, B). Interestingly, knocking down any one of these genes significantly prevented the coculture-induced upregulation of PAI-1, especially at the protein level (Fig. 7D), while knockdown of TGFβ1 and TGFBR1 produced a more obvious PAI-1 reduction at the transcriptional level (Fig. 7C). To further determine whether platelets can use the TGFβ/Smad signalling pathway to upregulate the expression of PAI-1 in cocultured cancer cells, we performed the following experiments. Here, cancer cells were treated with the TGFBR1 inhibitor SB431542, and we found that the mRNA levels of PAI-1 in B16F10-C3 and 231-GFP cells cocultured with platelets were significantly decreased by approximately 85% and 60%, respectively (Figure 7E, Fig. S5C). The protein levels of p-Smad2/3 and PAI-1 were also significantly decreased after SB431542 treatment (Figure 7F, Fig. S5D). SB431542 abolished the coculture-induced enhancement of cell migration in B16F10-C3 cells (Fig. 7G, Fig. S5E). Thus, we concluded that coculture with platelets promoted the malignancy of tumor cells by inducing TGFβ1 secretion and activating the TGFβ/Smad signalling pathway.

To determine how platelets protect cancer cells in circulation, we first detected some pro-survival proteins in B16F10-C3 and 231-GFP cells after coculture. Western blotting results showed that the levels of PAI-1 (6.7-fold), p-PI3K (6.4-fold), p-AKT at Ser473 (1.4-fold), p-AKT at Thr308 (4.3-fold), p-Bad (1.5-fold), and Bcl-2 (4.3-fold) were increased in the coculture group compared with the monoculture group of B16F10-C3 cells (Fig. 7H). To examine the relationship between the TGFβ-PAI-1 axis and the PI3K/AKT signalling pathway, we then determined the levels of these proteins in B16F10-C3 cells after knockdown of PAI-1. The results revealed that p-PI3K, p-AKT (Thr308), p-Bad and Bcl-2 were significantly reduced by 3-4-fold (Fig. 7H). Consistently, overexpression of PAI-1 elevated the protein levels of p-PI3K, p-AKT (Ser473 and Thr308), p-Bad and Bcl-2. Treatment with recombinant TGFβ1 protein also significantly elevated p-PI3K, p-AKT (Ser473 and Thr308), p-Bad and Bcl-2 levels.

One interesting finding is that between the two phosphorylation sites of AKT, coculture produced greater elevations of phosphorylation at Thr308 than at Ser473 (4.3-fold vs. 1.4-fold). Furthermore, overexpression of PAI-1 or addition of TGFβ also produced higher elevations of p-AKT308 than p-AKT473. Finally, knocking down PAI-1 dramatically decreased the level of p-AKT308 but had little effect on p-AKT437. All these observations indicate that the phosphorylation of AKT at Thr308 may be more relevant to coculture-mediated activation of the PI3K/AKT pathway in supporting melanoma cell survival in circulation.

In 231-GFP cells, we observed increased levels of PAI-1 (3.0-fold), p-PI3K (1.1-fold), p-AKT473 (2.3-fold), p-AKT308 (2.9-fold), p-Bad (1.9-fold) and Bcl-2 (2.1-fold) after coculture (Fig. S5F). Furthermore, a decrease in PAI-1 also reduced the levels of p-PI3K (1.2-fold), p-AKT473 (4.0-fold), p-AKT308 (1.8-fold), and p-Bad (2.3-fold) (Fig. S5F). Although treating 231-GFP cells with recombinant TGFβ1 protein significantly increased the levels of p-PI3K (1.5-fold), p-AKT308 (3.5-fold) and p-Bad (2.8-fold), it did not obviously alter the level of p-AKT473 (Fig. S5F). In summary, coculture of B16F10-C3 and 231-GFP cells with platelets increased the levels of pro-survival proteins such as p-PI3K, p-AKT (especially at Thr308), p-Bad, and Bcl-2 to promote cell survival in circulation through activation of the TGFβ/Smad/PAI-1 axis.

### PAI-1 is upregulated in malignant melanoma and TNBC

**Figure 8:**
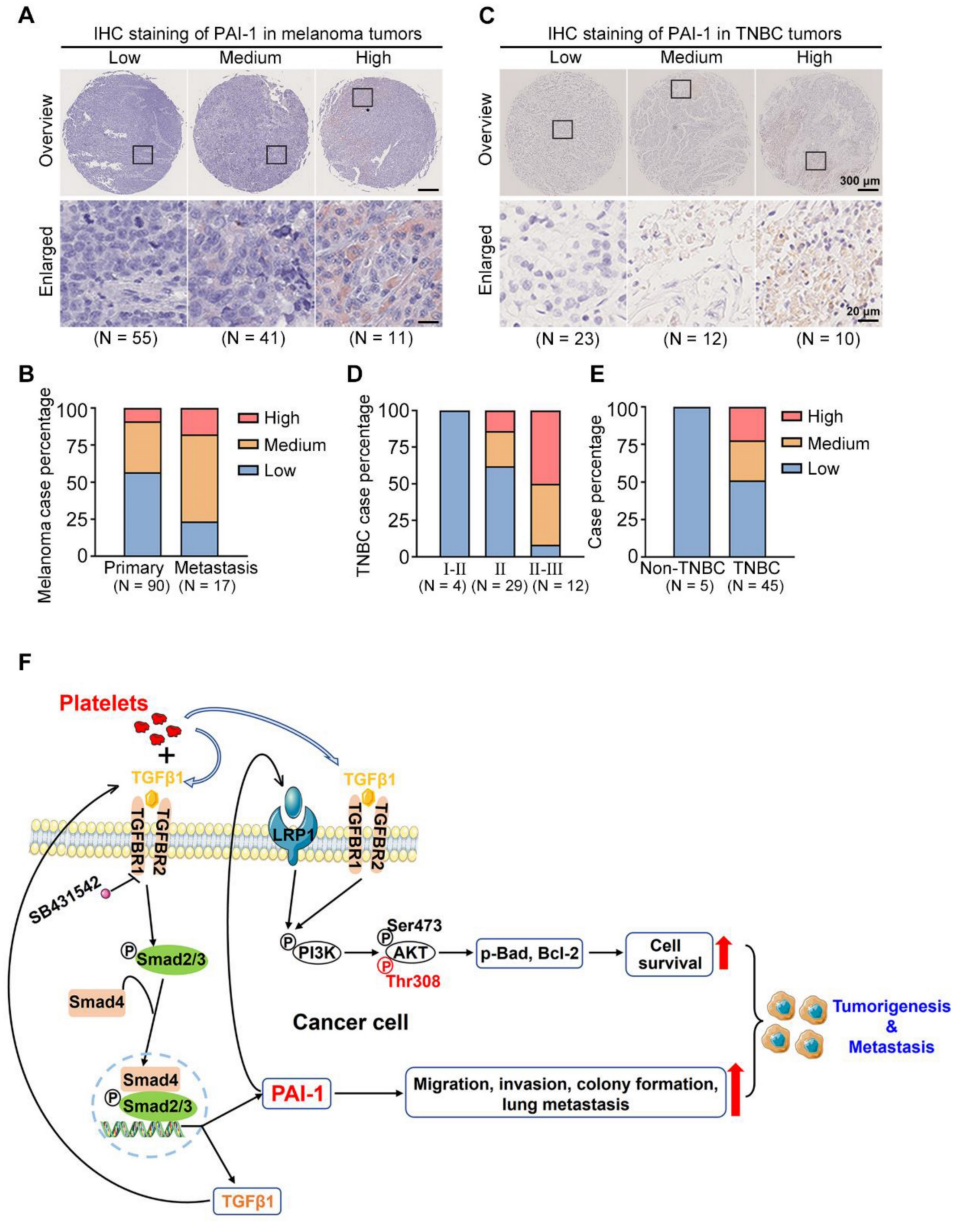
PAI-1 expression in clinical samples and the proposed signalling pathway. (**A**) PAI-1 expression in tumor samples derived from 107 melanoma patients was determined by IHC staining of a tissue microarray. Samples were sorted into several groups from low to high according to the staining intensity. (**B**) Percentages of patients with different expression levels of PAI-1 according to sample type. (**C**) IHC staining of PAI-1 in TNBC tumors. (**D**) TNBC case percentage from stages I to III based on the expression levels. (**E**) Case percentages of PAI-1 levels between the non-TNBC and TNBC groups. (**F**) The schematics illustrate the proposed signalling pathway through which coculture of tumor cells with platelets promotes cell survival, migration, invasion and colony formation capacities *in vitro* and tumorigenesis and metastasis in mice.

To assess the clinical importance of PAI-1 in tumor evolution, an IHC assay was performed to detect the levels of PAI-1 in 90 primary and 17 metastatic melanoma samples and 50 tumor samples (45 TNBC vs. 5 non-TNBC) and sorted them into low, medium, and high PAI-1 expression groups (Fig. 8A, C). For melanoma samples, the quantified results demonstrated that PAI-1 staining was significantly stronger in metastatic tumors than in primary tumors. The percentage of samples with high PAI-1 levels was approximately 2-fold higher in the metastatic tumors than in the primary tumors (Fig. 8B, Table S6). Similarly, TNBC patients in later stages also displayed higher PAI-1 levels. The percentage of samples with high PAI-1 expression was 3.6-fold higher in the stage II-III group than in the stage II group (Fig. 8D, Table S7). In addition, 50% of TNBC samples had medium to high levels of PAI-1, while 0% of non-TNBC samples had high levels of PAI-1 (Fig. 8E, Table S7).

In brief, our results illustrate that coculturing tumor cells with platelets increases the level of TGFβ1 (secreted by both platelets and tumor cells) in the culture medium. Then, TGFβ1 binds to TGFBR1/R2 in tumor cells to activate Smad2/3 signalling to increase the expression of PAI-1, which further activates PI3K/AKT^Thr308^, p-Bad and Bcl-2 to support tumor cell survival in circulation. In addition, high expression of PAI-1 induced by coculture increases the migration, invasion, colony formation and lung metastatic capacities of cancer cells. In this way, by interacting with platelets, tumor cells acquire the capacity to form larger tumors and tend to metastasize to distant organs (Fig. 8F).

## Discussion

Cancer cell survival is a prerequisite for cancer metastasis. Most CTCs are killed in the inefficient process of hematogenous metastasis before reaching distant organs. The mechanisms of cell survival in circulation are complicated. Undoubtedly, it is important to study the interactions between cancer cells and blood cells such as platelets.

Based on current knowledge, tumor cells adapt to survive in circulation by utilizing various mechanisms, including evasion of the immune system, expression of adhesion molecules, activation of survival pathways, formation of tumor microemboli and expression of chemokine receptors 52. The formation of microemboli requires various blood cells within clusters. During hematogenous metastasis, platelets have multifaceted effects on CTCs, and their interaction confers a strong stimulus for metastatic dissemination. In terms of detailed mechanisms, platelets can release VEGF, which stimulates the growth of blood vessels that supply tumour cells with oxygen and nutrients. In addition, platelets can secrete various cytokines and chemokines that can promote tumour cell growth, invasion, and metastasis. Pharmacological inhibition of platelets can also suppress tumour metastasis 53. The interacting molecules on CTCs, such as CD97, or those on host cells, such as integrins and P-selectin, can also aid in tumour cell survival and metastasis 54, 55.

In our previous study, we assessed the effects of shear stress and the expression of some important genes, including MnSOD, DSC2, and PKP1, on cell survival 2, 3. In this study, we wanted to further explore other components in the bloodstream, such as platelets. We found that monocultured B16F10-C3 cells were killed by circulatory treatment *in vitro*, and coculture of these cells with platelets greatly increased cell survival in circulation. The density of platelets used for this coculture model is also similar to that under physiological conditions, making our research more meaningful. We found that after being cocultured with platelets, cancer cells displayed stronger metastatic abilities. Specifically, cocultured B16F10-C3 and 231-GFP cells showed enhanced migration, invasion, colony formation, experimental lung metastasis, tumorigenesis, and metastasis. Thus, platelets could be studied as an important hematogenous factor contributing to tumor metastasis. After observing the above phenotypic changes, we found that SERPINE1 was effectively upregulated in cocultured cells through RNA-seq of both B16F10-C3 and 231-GFP cells.

SERPINE1 encodes PAI-1 and is mainly produced by various sources, such as endothelial cells, liver, kidneys, adipose tissue, cancer cells and platelets 56, 57. PAI-1 expression can be regulated by many modulators, such as growth factors and cytokines, which are considered tumor-promoting factors 58. PAI-1 has multifaceted activities in human cancer: it sustains proliferative signals; protects against cell death; and promotes angiogenesis, invasion, metastasis and tumor-promoting inflammation 59-62. It protects against cell death by suppressing intrinsic apoptosis via caspase-3, enhancing cell migration, increasing adherence to fibronectin, attenuating apoptosis via inhibition of FasL-mediated apoptosis, protecting against apoptosis by stimulating cJun/ERK and elevating prosurvival proteins such as Bcl-2 and Bcl-XL. However, the effects of PAI-1 in increasing *in vivo* metastasis and the mechanism are not clearly defined 62. Therefore, exploring the new mechanisms of PAI-1 in cancer cell survival and metastasis is still necessary.

We further validated the role of PAI-1 in cell survival and metastasis. Knocking down PAI-1 attenuated the promotion of cancer cell survival in circulation, tumorigenesis and metastasis induced by coculture with platelets. Moreover, overexpression of PAI-1 enhanced the survival of B16F10-C3 cells in circulation, tumorigenesis and metastasis. TGFβ, the major transcriptional activator of PAI-1, regulates the synthesis of PAI-1 mRNA via its major downstream transcription factors (SMADs).

As a TGFβ-responsive gene, SERPINE1 plays a significant role in a variety of processes, such as signal transduction, tumor growth and metastasis. During intravasation, platelets can be recruited and activated. Platelets contain many factors, such as TGFβ, VEGF, and bFGF. In our RNA-seq data, we found that TGFβ-related genes were increased only in B16F10-C3 cells, and their protein levels were increased after coculturing in both melanoma and TNBC cells, while no obvious changes were observed for factors such as VEGF and bFGF from the RNA-seq results. Furthermore, TGFβ/Smad signalling can be activated in tumor cells upon platelets exposure 31. Therefore, we chose TGFβ1 as the upstream gene of SERPINE1 for signalling pathway analysis. In our coculture system, platelets promoted the survival and metastasis of malignant tumor cells by activating the TGFβ/Smad and PI3K/AKT pathways.

During tumorigenesis, protein kinase B, also called AKT, is often over-activated in tumor cells, leading to strengthened proliferation, growth, and survival 63. AKT is activated mainly in two ways: the protein kinase pyruvate dehydrogenase kinase 1 (PDK1) phosphorylates the threonine 308 site (Thr308), and the mTORC2 complex activates the serine 473 site (Ser473) 64, 65. The Ser473 site has been widely studied 66, 67, while phosphorylation at Thr308 remains incompletely understood 68. Thr308 activation has been correlated with an important event of AKT activation 64. In head and neck squamous cell carcinoma cell lines, PAI-1 expression was closely correlated with the activation status of the PI3K/AKT signalling pathway by detecting AKT^Thr308^ activity 69. Sakamoto reported that recombinant human PAI-1 treatment can activate AKT signalling by phosphorylation at the Thr308 site to promote oesophageal squamous cell carcinoma invasion via LRP1 70. Although the TGFβ/Smad and PI3K/AKT pathways are well studied in cancer progression, we found a new link between PAI-1 and p-AKT (Thr308) when melanoma and TNBC cells acquired stronger survival and metastatic abilities after being cocultured with platelets. The supporting evidence includes that the levels of PAI-1 and p-AKT (Thr308) were both elevated after coculture. Second, the level of p-AKT (Thr308) was greatly enhanced when PAI-1 was overexpressed in B16F10-C3 cells.

In this study, we also observed that high expression of PAI-1 was associated with a higher degree of malignancy in melanoma and higher tumor grade in TNBC patients; high levels of PAI-1 were also associated with worse OS and DMFS in breast cancer patients. Last, as the bloodstream is a complicated system, the advantage of our study is that we used two different sources of platelets, one from mice and another from humans, to verify our results. One limitation of our coculture model is that it cannot completely represent the true environment that CTCs encounter in blood circulation, such as red and white blood cells, which could also exert influence on CTCs to alter their survival and metastasis. In the future, we will continue to study the role of shear stress on the interactions between CTCs and hematocytes.

## Conclusion

In summary, the findings from this study revealed PAI-1 as a key mediator through which platelets can strengthen the survival and metastatic abilities of cancer cells. During coculture, PAI-1 can be upregulated through the TGFβ/Smad pathway, it can then activate the PI3K/AKT signalling pathway. Our results suggest that PAI-1 may act as a potential biomarker for detecting and targeting metastatic tumor cells.

## Supplementary Material

Supplementary figures and tables.
