# Supplementary material for: Coculture of cancer cells with platelets increases their survival and metastasis by activating the TGFβ/Smad/PAI-1 and PI3K/AKT pathways
Source: Int J Biol Sci. 2023 Aug 15;19(13):4259–77. doi: 10.7150/ijbs.85986 (PMC10496510; doi:10.7150/ijbs.85986)
Supplement: Supplementary file 1 — Supplementary figures and tables. [file ijbsv19p4259s1.pdf]

Coculture of cancer cells with platelets increases their survival and metastasis by activating the TGFβ/Smad/PAI-1 and PI3K/AKT pathways

Haibo Tong[1], Koukou Li[1], Muya Zhou[1], Renfei Wu[1], Hongmei Yang[1], Zheng Peng[1], Qi Zhao[1,2], Kathy Qian Luo[1,2*]

[1]Faculty of Health Sciences, University of Macau, Taipa, Macao SAR, China.

[2]Ministry of Education Frontiers Science Center for Precision Oncology, University of Macau.

*Corresponding author: Kathy Qian Luo; E-mail: kluo@um.edu.mo

Supplementary Figures

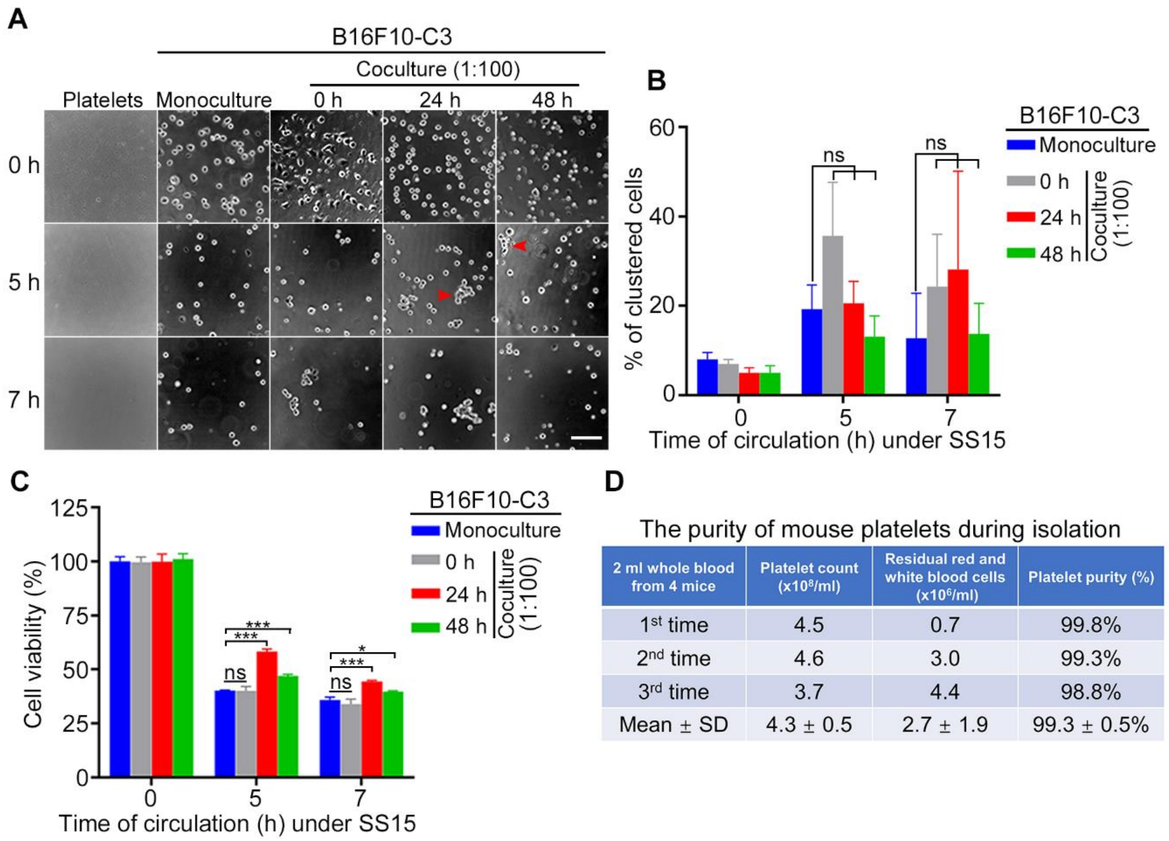

The purity of mouse platelets during isolation

2 ml whole blood from 4 mice	Platelet count (x10⁸/ml)	Residual red and white blood cells (x10⁶/ml)	Platelet purity (%)
1st time	4.5	0.7	99.8%
2nd time	4.6	3.0	99.3%
3rd time	3.7	4.4	98.8%
Mean ± SD	4.3 ± 0.5	2.7 ± 1.9	99.3 ± 0.5%

Fig. S1. Results for B16F10-C3 cells cocultured with platelets at a 1:100 ratio under SS15 treatment. (**A**) Representative phase contrast images of mouse platelets, monocultured and cocultured cells (cocultured with murine platelets at 1:100 for different time points) after 0, 5 and 7 h of SS treatment. The red arrowhead indicates clustered cells. Scale bar, 100 µm. (**B**) Quantified % of clustered cells. (**C**) Cell viability was determined by the MTT assays. The data are shown as the mean ± SD. (**D**) The purity of mouse platelets during isolation.

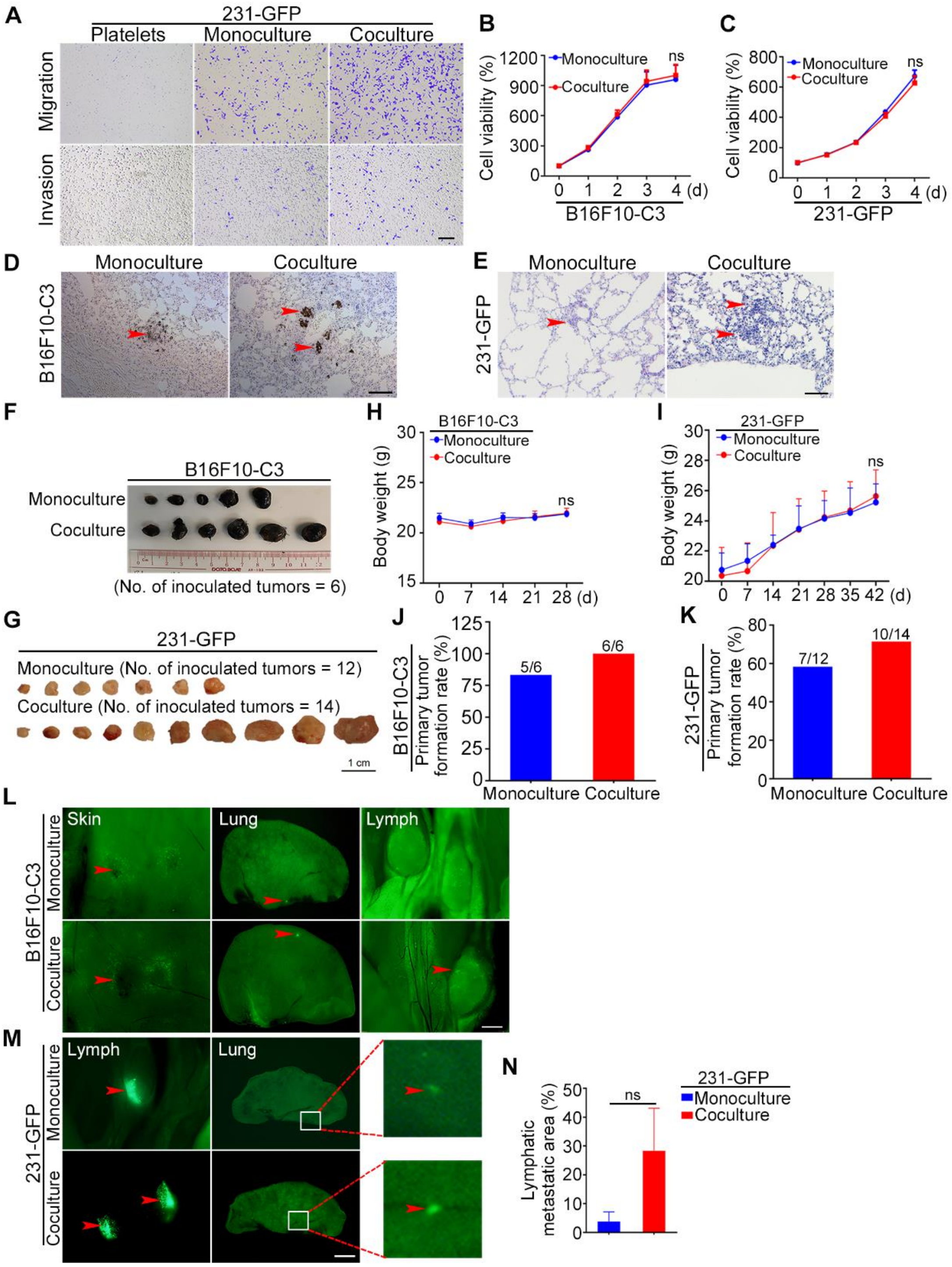

Fig. S2. Coculture induced tumorigenesis and metastasis in orthotopic tumor models. (**A**) Representative image of the Transwell migration and invasion assays for human platelets, monocultured and cocultured 231-GFP cells. Scale bar, 200 µm. (**B** and **C**) MTT assays were used to compare cell proliferative ability between monocultured and cocultured B16F10-C3 and 231-GFP cells. (**D** and **E**) H&E staining images of lung tissues under the indicated conditions. Scale bar, 20 µm. (**F** and **G**) Images of the primary tumors inoculated with monocultured and cocultured B16F10-C3 (No. of mice = 6) and 231-GFP cells (No. of mice = 6 to 7). (**H** and **I**) Quantification of mouse body weight. The results represent the mean ± SEM.

(**J** and **K**) Quantified results of the primary tumor formation rate. (**L** and **M**) Images of metastatic tumors located in different organs in B16F10-C3 and 231-GFP cells (No. of mice = 6 to 7). The white box shows the enlarged view. The red arrowhead indicates metastatic tumors. Scale bars, 1 mm. (**N**) Quantified results of the iliac lymphatic metastasis area in 231-GFP cells. The data are shown as the mean ± SD. ns, no significance.

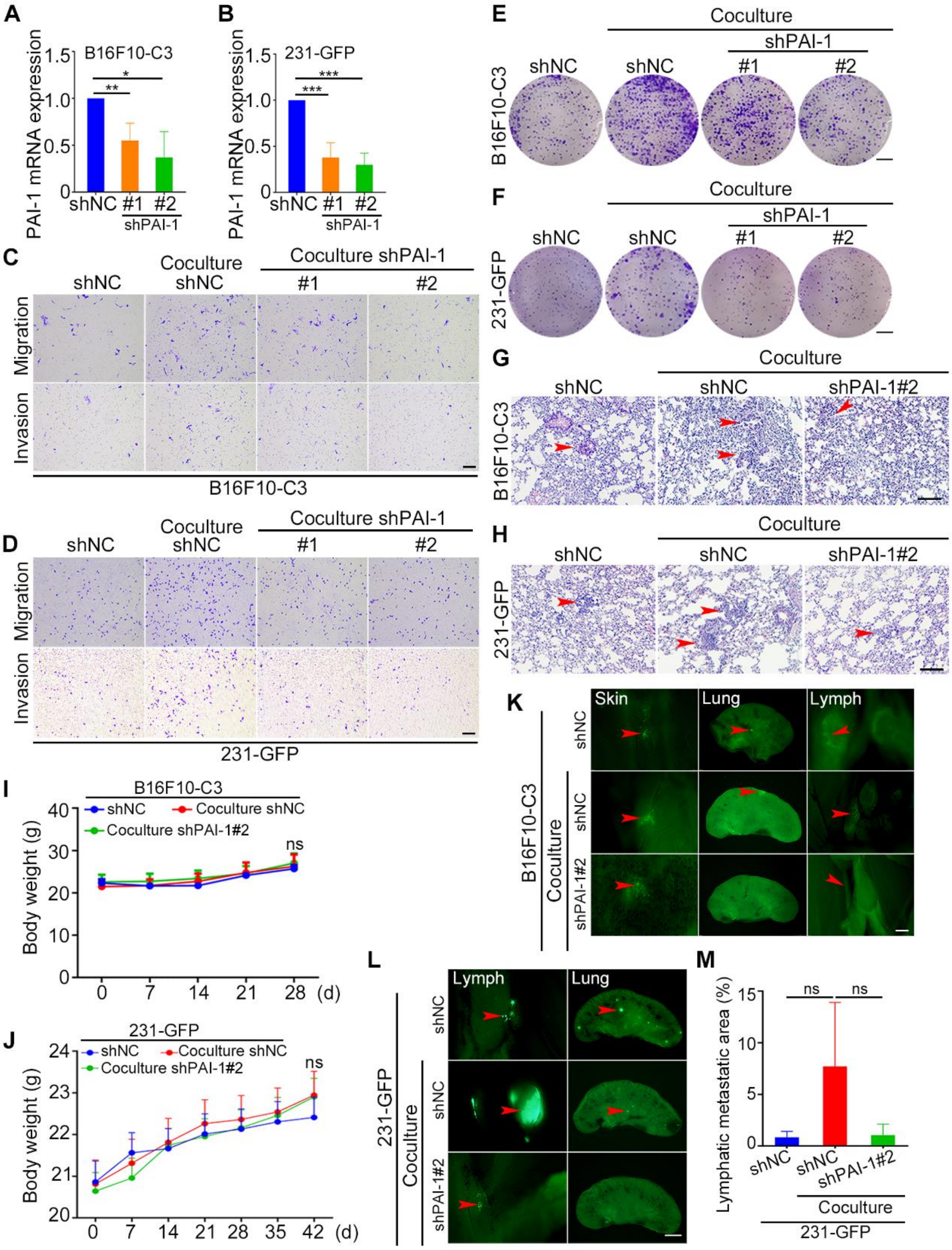

Fig. S3. Knockdown of PAI-1 effectively reduced the tumorigenesis and metastasis abilities of B16F10-C3 and 231-GFP cells. (**A** and **B**) qRT-PCR results showing the knockdown efficiency of PAI-1 by using shRNAs in B16F10-C3 and 231-GFP cells. (**C** and **D**) Transwell migration and invasion assays for monocultured shNC, cocultured shNC and cocultured shPAI-1#1 and shPAI-1#2 B16F10-C3 and 231-GFP cells. Scale bar, 200 μm. (**E** and **F**) Colony formation assay for the monocultured shNC, cocultured shNC and cocultured shPAI-1#1, shPAI-1#2 of B16F10-C3 and 231-GFP cells. Scale bar, 5 mm. (**G** and **H**) H&E staining assays of lung lobes inoculated with the indicated cells. Scale bar, 20 μm. (**I** and **J**) Quantified results of mouse

body weight after knocking down PAI-1 in B16F10-C3 and 231-GFP cells. (**K** and **L**) Representative fluorescent images of metastatic tumors located in different organs after injection of B16F10-C3 (No. of mice = 5) and 231-GFP cells (No. of mice = 7 to 8) with PAI-1 knockdown. The red arrowhead indicates metastatic tumors. Scale bars, 1 mm. (**M**) Quantified results of the iliac lymphatic metastasis area in 231-GFP cells. The data are shown as the mean ± SD. *$P < 0.05$, **$P < 0.01$, ***$P < 0.001$ and ns, no significance.

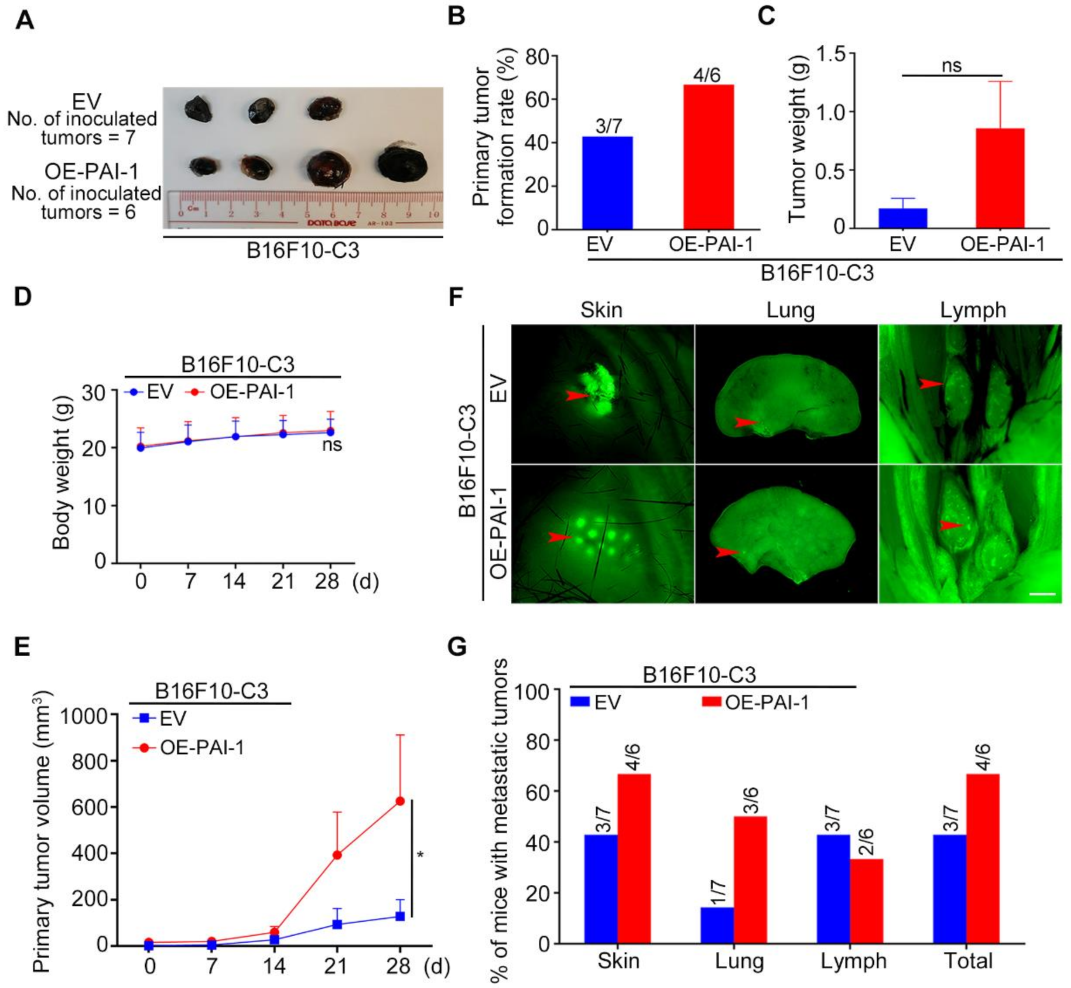

Fig. S4. Overexpression of PAI-1 promoted B16F10-C3 cell tumorigenesis and metastasis. (**A**) Representative image of the primary tumors after subcutaneous injection of B16F10-C3 cells into C57BL/6J mice (n = 6 to 7). (**B** and **C**) Quantification of the primary tumor formation rate and tumor weight of EV and OE-PAI-1 in B16F10-C3 cells. The results represent the mean ± SEM. (**D**) Quantified results of mouse body weight. (**E**) Quantified results of primary tumor volume. The results represent the mean ± SEM. (**F**) Representative images of metastatic tumors in the distant skin, lung, and iliac lymph nodes in the EV and OE-PAI-1 groups. The red arrowhead indicates metastatic tumors. Scale bars, 1 mm. (**G**) Quantification of the metastatic rate in different organs in mice in the EV and OE-PAI-1 groups. *$P < 0.05$ and ns, no significance.

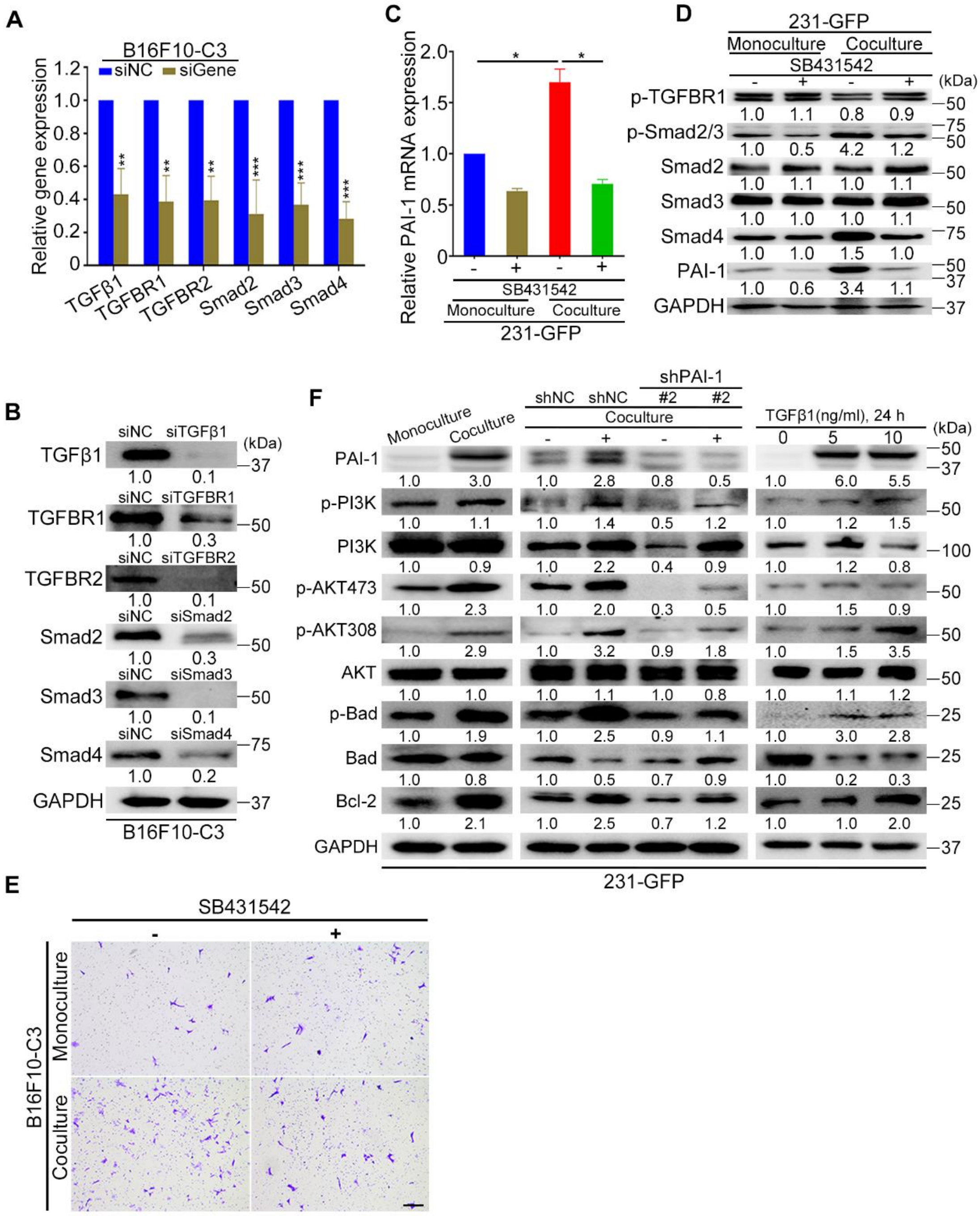

Fig. S5. Elucidating the signalling pathways of PAI-1. (**A** and **B**) mRNA and protein levels of target gene expression using siRNA treatment. (**C**) mRNA level of PAI-1 in 231-GFP cells after SB431542 treatment. (**D**) Protein levels of p-TGFBR1, p-Smad2/3, Smad2-4, and PAI-1 after SB431542 treatment in 231-GFP cells. (**E**) Transwell migration assay of B16F10-C3 cells after SB431542 treatment. Scale bar, 200 μm. (**F**) Protein levels of the PI3K/AKT survival pathway between monocultured and cocultured, knockdown of PAI-1 and recombinant TGFβ1 protein treatment in 231-GFP cells. The data are shown as the mean ± SD. **P* < 0.05, ***P* < 0.01, ****P* < 0.001 and ns, not significant.

Supplementary tables

Table S1 List of primers for qRT-PCR.

Primer name	Sequence information (5’-3’)
M-SERPINE2-F	ACATGGGATCGCGTCCATC
M-SERPINE2-R	GCCACGGTCACAATGTCTTT
M-PAI-1-F	TTCAGCCCTTGCTTGCCTC
M-PAI-1-R	ACACTTTTACTCCGAAGTCGGT
H-PAI-1-F	CCTGGGCACTTACAGGAAGG
H-PAI-1-R	GGTCCGATTCGTCGTCAAATAAC
M-GAPDH-F	AATGGATTTGGACGCATTGGT
M-GAPDH-R	TTTGCACTGGTACGTGTTGAT
H-GAPDH-F	AGCCACATCGCTCAGACA
H-GAPDH-R	GCCCAATACGACCAAATCC
M-TGFβ1-F	CTCCCGTGGCTTCTAGTGC
M-TGFβ1-R	GCCTTAGTTTGGACAGGATCTG
M-TGFBR1-F	TCTGCATTGCACTTATGCTGA
M-TGFBR1-R	AAAGGGCGATCTAGTGATGGA
M-TGFBR2-F	CCGCTGCATATCGTCCTGTG
M-TGFBR2-R	AGTGGATGGATGGTCCTATTACA
M-Smad2-F	ATGTCGTCCATCTTGCCATTC
M-Smad2-R	AACCGTCCTGTTTTCTTTAGCTT
M-Smad3-F	CACGCAGAACGTGAACACC
M-Smad3-R	GGCAGTAGATAACGTGAGGGA
M-Smad4-F	ACACCAACAAGTAACGATGCC
M-Smad4-R	GCAAAGGTTTCACTTTCCCCA
H-TGFβ1-F	CAATTCCTGGCGATACCTCAG
H-TGFβ1-R	GCACAACTCCGGTGACATCAA
H-TGFBR1-F	GCTGTATTGCAGACTTAGGACTG
H-TGFBR1-R	TTTTTGTTCCCACTCTGTGGTT
H-TGFBR2-F	AAGATGACCGCTCTGACATCA
H-TGFBR2-R	CTTATAGACCTCAGCAAAGCGAC
H-Smad2-F	CCGACACACCGAGATCCTAAC
H-Smad2-R	GAGGTGGCGTTTCTGGAATATAA
H-Smad3-F	TGGACGCAGGTTCTCCAAAC
H-Smad3-R	CCGGCTCGCAGTAGGTAAC
H-Smad4-F	CCACCAAGTAATCGTGCATCG
H-Smad4-R	TGGTAGCATTAGACTCAGATGGG

Table S2 List of shRNA and siRNA.

shRNA name	TRC number	Sequence information (5'-3')
M-PAI-1#1		CCCTCTACTTCAGTGGCCAAT
M-PAI-1#2	0000305135	AGTGGAAAGAGCCAGATTTAT
H-PAI-1#1	0000331004	TCTCTGCCCTCACCAACATTC
H-PAI-1#2	0000331006	AGACCAACAAGTTCAACTATA
shCtrl	SHC002	CAACAAGATGAAGAGCACCAA
M-siTGFβ1		ACCCCCACUGAUACGCCUGAGTT
M-siTGFBR1		GAAUUAAAAAAACAUUGUCACTT
M-siTGFBR2		CGCCAACAACAUCAACCACAATT
M-siSmad2		AUCGCAUACUAUGAACUAAACTT
M-siSmad3		AAUAUUCCAGAAACCCCACCUTT
M-siSmad4		CUACCCAAGCGCGUAUAUAAATT
siCtrl		UUCUCCGAACGUGUCACGUTT

Table S3 List of overexpression plasmids.

Vector name	Transcript ID	Sequence length
M-PAI-1	NM_008871.2	1209 bp

Table S4 Analysis of primary tumor formation of B16F10-C3 cells in orthotopic tumor models.

Group	With tumor	Without tumor	P value
Monoculture	5	1	0.9999[a]
Coculture	6	0	
shNC	3	2	0.0357[a]
Coculture shNC	5	0	
Coculture shPAI-1#2	1	4	
EV	3	4	0.5921[a]
OE-PAI-1	4	2	

[a]Chi-square test p value is indicated.

Table S5 Analysis of primary tumor formation of 231-GFP cells in orthotopic tumor models.

Group	With tumor	Without tumor	P value
Monoculture	7	5	0.6828[a]
Coculture	10	4	
shNC	9	7	0.0042[a]
Coculture shNC	10	6	
Coculture shPAI-1#2	1	13	

[a]Chi-square test p value is indicated.

Table S6 Correlation of PAI-1 expression with clinicopathological features of melanoma patients.

	**PAI-1**				
	**Negative**	**Positive**	**Positive ratio (%)**	**Total**	**P value**
**Sample types**					
Primary	51	39	43.3	90	0.0165[a]
Metastasis	4	13	76.5	17	
Total	55	52	48.6	107	
**Lymph node involvement**					
Negative	54	40	42.6	94	0.2411[a]
Positive	5	8	61.5	13	
Total	59	48	44.8	107	
**Age (mean ± SD)**	63.8 ± 13.2	60.2 ± 15.4			0.1999[b]
Age (range)	30–82	29–92			

Table S7 Correlation of PAI-1 expression with clinicopathological features of TNBC patients.

	**PAI-1**				
	**Negative**	**Positive**	**Positive ratio (%)**	**Total**	**P value**
**Grade**					
I-II	4	0	0.0	4	0.0214[a]
II	25	4	13.8	29	
II-III	6	6	50	12	
Total	35	10	22.2	45	
**Lymph node involvement**					
Negative	25	10	28.6	35	0.089[a]
Positive	10	0	0.0	10	
Total	35	10	22.2	45	
**Age (mean ± SD)**	46.1 ± 12.8	53.8 ± 13.7			0.126[b]
Age (range)	29-90	27-72			
**Size (mean ± SD, cm)**	3.4 ± 1.3	3.7 ± 1.2			0.516 [b]
Size (range)	0.4-6.7	1.2-5.3			

[a]Chi-square test p value is indicated.

[b]Age and tumor size were calculated by unpaired Student's t test.
